# Requirements for designing cluster randomised control trials to detect suppression of malaria vector population densities

**DOI:** 10.1186/s12915-025-02414-5

**Published:** 2025-10-09

**Authors:** Penelope A. Hancock, Tin-Yu J. Hui, Patric S. Epopa, Azize Milogo, Andrew R. McKemey, Franck A. Yao, Abdoulaye Diabaté, Austin Burt

**Affiliations:** 1 Department of Infectious Disease Epidemiology, Imperial College London, London, UK; 2 Department of Life Sciences, Imperial College London, London, UK; 3 Institut de Recherche en Sciences de La Santé/Centre Muraz, Bobo-Dioulasso, Burkina Faso; 4 Institut Des Sciences Des Sociétés, Ouagadougou, Burkina Faso

**Keywords:** Cluster randomised control trials, Gene drive, Geostatistical model, Malaria, Vector control, Sterile insect technique, Genetically modified organism, Mosquito-borne disease

## Abstract

**Background:**

Novel interventions for mosquito-borne disease control which release modified mosquitoes that are sterilised or genetically modified to cause offspring inviability are progressing towards field applications. Cluster randomised control trials (CRCTs) could provide robust assessment of intervention efficacy in suppressing mosquito populations in field environments, but guidance on designing CRCTs to detect mosquito suppression impacts is limited.

**Results:**

We developed statistical models to simulate CRCTs, informed by a 5-year time series measuring densities of malaria vector species from the *Anopheles gambiae* complex in four villages in western Burkina Faso. We estimated requirements for parallel and step wedge designs, varying the targeted vector species, the suppression effect and the monitoring regime. For a suppression effect of 50%, 21–22 clusters were required to detect suppression with 90% power when all *An. gambiae* complex species were targeted, while 24–26 clusters were required when only *An. coluzzii* was targeted and 60–66 clusters were required when only *An. gambiae* was targeted. For stronger suppression effects, required trial sizes depended less on target species, with 9–10 clusters being sufficient to detect a 90% suppression effect. We investigated how reducing sampling effort, by sampling fewer houses and restricting sampling to rainy season months, affected statistical power.

**Conclusions:**

Our results provide empirically based guidance for designing CRCTs to evaluate interventions aiming to suppress malaria vector populations.

**Supplementary Information:**

The online version contains supplementary material available at 10.1186/s12915-025-02414-5.

## Background

Many strategies for controlling mosquito disease vectors act by suppressing vector populations. Interventions that use chemical insecticides to target adult mosquito life stages, including insecticide treated bednets (ITNs), indoor residual spraying (IRS) and outdoor ‘space’ spraying, have played a critical role in disease control over several decades [1, 2], and remain important today. These interventions are, however, currently threatened by the widespread emergence of insecticide resistance in vector populations [3], and thus a wider range of vector control tools is needed to achieve progress in mosquito-borne disease control and elimination, including novel technologies [4].

Strategies for suppressing vector populations involving field releases of modified adult male mosquitoes are increasingly being developed and deployed. Releases of males which are sterilised through irradiation [5], or genetically modified such that their offspring are inviable [6], have been trialled in a number of global regions since the 1970s [5, 7, 8]. Recently, in Singapore, releases of male *Ae. aegypti* mosquitoes infected with a strain of *Wolbachia* bacteria that causes matings with uninfected females to produce inviable offspring have achieved substantial reductions in dengue cases [9].

To effectively suppress vector populations, the above strategies require large numbers of males to be reared in mass rearing facilities and released [9, 10]. This issue may be overcome by novel approaches where modified genes are inherited across successive generations of the mosquito population following release, causing more sustained suppression. A suite of approaches with diverse underlying genetic mechanisms, technological implementations and potential impacts on mosquito populations have been proposed [11–14]. Some strategies are ‘self-limiting’ [11], whereby the released genes persist for a small number of generations before being lost from the population. For example, the Friendly™ technology developed by Oxitec, which produces genetically modified (GM) male mosquitoes whose matings with wild females produce inviable female offspring (male offspring survive), has recently been released in Djibouti in the invasive malaria vector *An. stephensi* [15]*.* At the other end of the spectrum, ‘low-threshold’ gene drive technologies are being investigated [11], whereby the released genes could potentially increase in frequency across successive generations and spread indefinitely throughout populations of the targeted mosquito species [16]. For instance, a gene drive that confers sterility in homozygous females has been shown to spread and induce the complete suppression of large cage populations of the malaria vector *Anopheles gambiae* [17]. Field trials of low-threshold gene drives are currently being actively considered [18].

Compared to mainstay insecticide-based interventions like ITNs and IRS, the efficacy of mosquito release strategies for vector population suppression, such as SIT, self-limiting GM mosquito releases and gene drive releases, is not yet well understood. Cluster randomised control trials (CRCTs) are the gold standard methodology for robust quantification of the efficacy of public health interventions that impact disease outcomes across whole communities [19] and are commonly used to support applications for World Health Organisation (WHO) approval for intervention rollout [20]. CRCTs randomise the allocation of an intervention across a series of communities, or clusters, with those not receiving the intervention acting as controls [19]. Several CRCTs have been conducted to promote the scaling up of chemical insecticide-based interventions for vector control [21–24], but most trials of mosquito release interventions for population suppression have been smaller scale pilot trials [7]. More recently, larger trials releasing *Wolbachia-*infected male mosquitoes compared several treatment and control locations and demonstrated substantial impacts on vector suppression and disease incidence, although the designs did not randomise the allocation of treatment and control sites [9, 25].

Mosquito release interventions target wild populations of particular vector species by rearing and releasing modified mosquitoes of these species. The majority of trials conducted thus far have investigated mosquito releases in the *Aedes* genus, which transmit arboviruses such as the dengue, Zika, yellow fever and chikungunya viruses. Relatively few trials have conducted mosquito releases in *Anopheles* malaria vector species [7]. In Africa, malaria vectors include seven species from the *An. gambiae* complex [26, 27], as well as *An. funestus* and the invasive species *An. stephensi,* with cryptic species also implicated in transmission [28]. Epidemiological impacts will therefore depend on the abundance of the targeted vector species relative to that of other non-target vector species present in the area [29]. Thus, we need to understand the direct impacts of mosquito release interventions in suppressing populations of targeted vector species to estimate their potential efficacy and how this varies across different environments.

There is a lack of CRCTs of malaria vector control interventions that consider vector population suppression as their primary endpoint, although several have assessed suppression as a secondary endpoint [30–32]. Vector densities can be highly variable [33, 34], logistically intensive to monitor and dependent on the collection method [35]. Potentially high variability in the densities of the vector species targeted by the intervention may mean that large trial sizes are required to robustly detect vector population suppression effects with sufficient statistical power [19].

Large CRCTs for novel mosquito release interventions may be logistically challenging, requiring significant stakeholder engagement which could be difficult to coordinate when releasing in many clusters simultaneously. However, for interventions where modified genes persist in wild mosquito populations following releases, smaller pilot trials could still affect large areas if the modified genes diffuse spatially [18]. Step wedge CRCT designs, where the initiation of the intervention is staggered such that some clusters receive the intervention later than others, could possibly enable robust assessment of intervention efficacy while allowing the intervention to initially be applied in a small number of clusters.

To support the design of trials to evaluate novel mosquito release interventions for malaria control, we develop statistical models to simulate data obtained from CRCTs aiming to detect suppression in the numbers of adult *Anopheles* malaria vectors resulting from a vector control intervention. Our models are informed by a 5-year time series of malaria vector density measurements obtained using pyrethrum spray catch (PSC) methods conducted in four villages in south west Burkina Faso, which recorded densities of three major vector species from the *An. gambiae* complex (*An. gambiae*,* An. coluzzii* and *An. arabiensis*)*.* Mosquitoes were collected in all months of the year, typically at 20 geolocated houses per village, allowing spatiotemporal variability in mosquito counts to be characterised. We compare simulated CRCTs where different vector species are targeted by the intervention, considering a range of trial designs, suppression effect sizes and monitoring intensities. Our results provide empirically based estimates of statistical power that can inform the design of upcoming field trials aiming to obtain robust estimates of malaria vector population suppression impacts.

## Results

### Simulating mosquito counts across space and time

We fitted a spatiotemporal geostatistical model to counts of mosquito species from the *An. gambiae* complex collected by PSC from each house, village and month over a 5-year period covering July 2012 to July 2014 and January 2017 to December 2019. Collections were made from four villages all within 30 km of Bobo Dioulasso: Bana centre, Bana market, Pala and Souroukoudingan [36]. Typically, 20 houses per month were sampled in Bana centre, Pala and Souroukoudingan, and 6 houses per month were sampled in the smaller settlement of Bana market, with missing records in some months (see the “Methods” section).

**Fig. 1:**
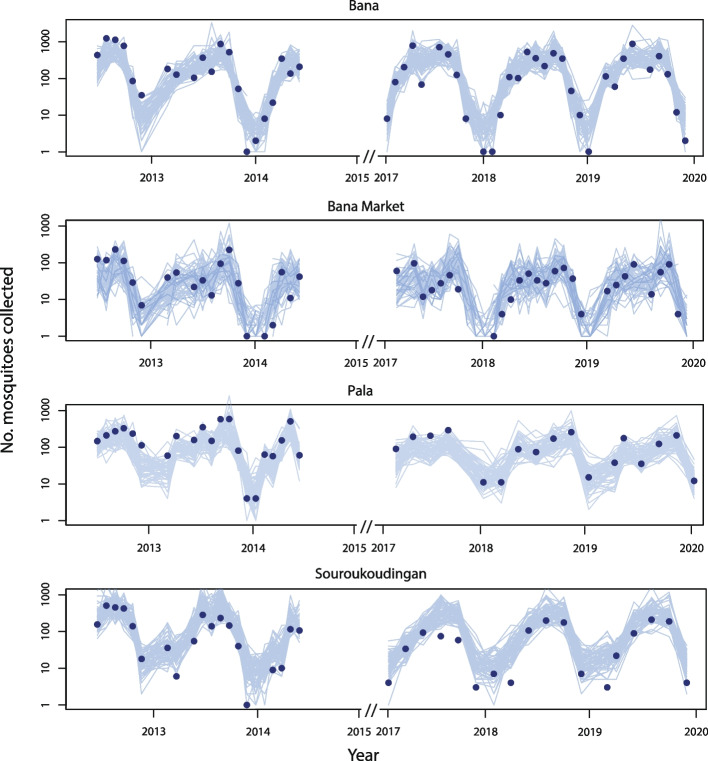
Posterior samples of the predicted number of mosquitoes collected in each month, year and village compared to observed values. Lines show sixty random draws from the posterior distribution predicted by the geostatistical model, and markers show the data on numbers of mosquitoes collected. The geostatistical model (Eq. 1) has 5 fitted parameters

**Fig. 2:**
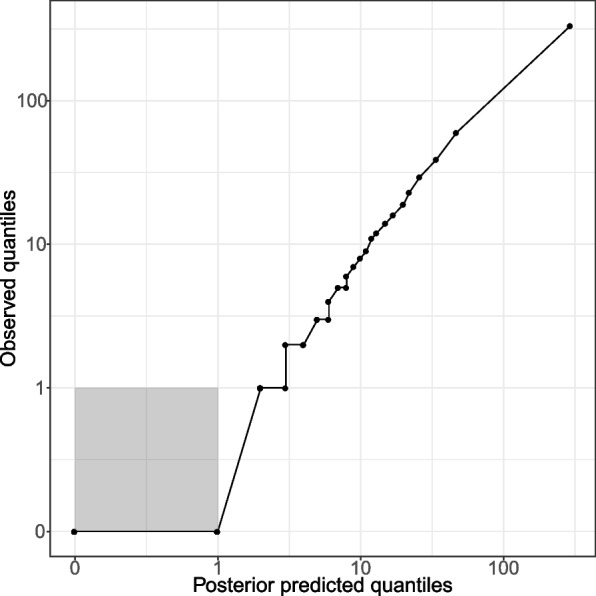
A Q-Q plot showing the quantiles of the observed total mosquito counts versus the quantiles of the posterior mean fitted values of the mosquito counts in each house, month and village. Black circles show the sequence of 41 quantiles increasing from zero to one at evenly spaced intervals of 0.025. The grey shaded region shows the area of the graph corresponding to a linear scale on both axes, with the remaining area showing a log scale on both axes

Simulations from the posterior of the fitted model (see the “Methods” section) captured monthly and seasonal variation in the total number of mosquitoes caught in each village (Fig. 1). The posterior predictive distribution of the mosquito counts per house agreed well with the observed distribution (Fig. 2). The fitted geostatistical model indicated significant overdispersion of mosquito counts across both houses and villages (Table 1). Significant spatial and temporal autocorrelation was also identified, notably with negative temporal autocorrelation in mosquito counts across months (Table 1). Predictive maps generated by the geostatistical model suggest that the locations where high mosquito counts occur are not consistent over time (Additional file 1: Figs. S6, S7 and S8) [37–40], with locations tending to switch from having high counts to low counts between consecutive months (Additional file 1: Fig. S9).

**Table 1:** Parameters of the spatiotemporal geostatistical model fitted to the counts of mosquitoes collected by PSC

Parameter	Mode	0.025% CI	97.5% CI
Range of spatial autocorrelation (*r*)	0.0018 (° N)	0.0016 (° N)	0.0021 (° N)
Standard deviation of spatially-correlated random effect (${\sigma }_{\omega }^{2}$)	4.83 ($\text{log}(no. mosquitoes)$)	4.17 ($\text{log}(no. mosquitoes)$)	5.90 ($\text{log}(no. mosquitoes)$)
Monthly temporal autocorrelation ($\rho$)	− 0.67	− 0.78	− 0.54
Standard deviation of the house level random effect (${\nu }_{h}$)	0.71 ($\text{log}(no. mosquitoes)$)	0.66 ($\text{log}(no. mosquitoes)$)	0.77 ($\text{log}(no. mosquitoes)$)
Standard deviation of the village level random effect (${\mu }_{v}$)	1.0 ($\text{log}(no. mosquitoes)$)	0.88 ($\text{log}(no. mosquitoes)$)	1.14 ($\text{log}(no. mosquitoes)$)

**Fig. 3:**
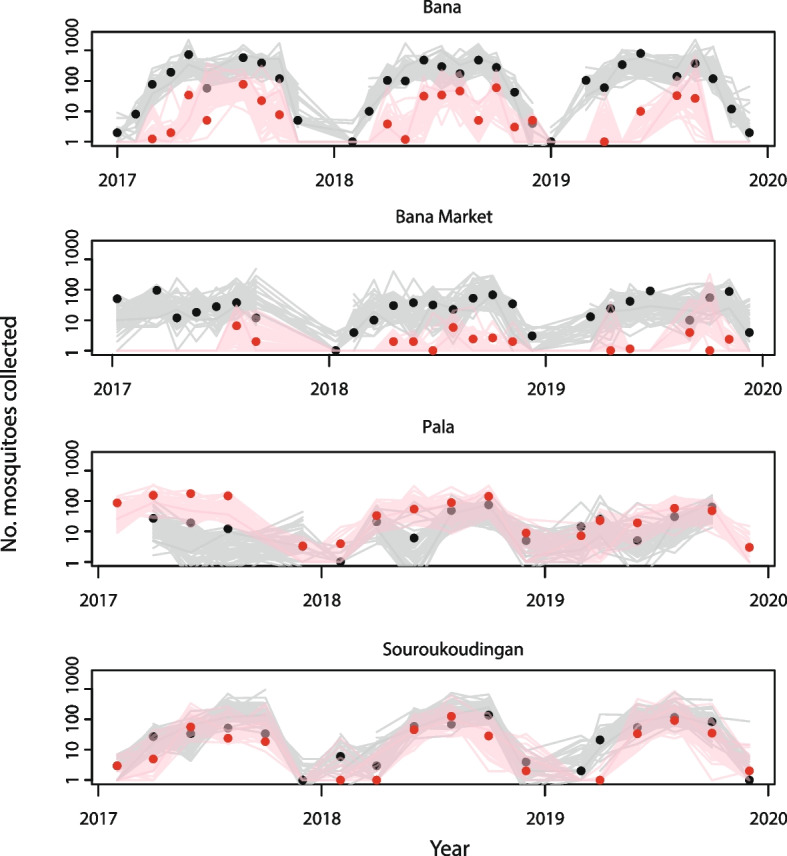
Posterior samples of the predicted number of mosquitoes of each species collected in each month, year and village compared to observed values. Black lines show sixty random draws from the posterior distribution of the numbers of *An. coluzzii* predicted by the geostatistical model (Eq. 5) and black markers show the expected value of the number collected (${p}_{hmv}^{c}{y}_{hmv}$). Red lines and markers show corresponding values for the numbers of *An. gambiae* collected. The geostatistical model (Eq. 1) has 5 fitted parameters

Molecular identification of collected *An. gambiae* complex mosquito species was performed in the years 2017–2019 only (see the “Methods” section). The vast majority (93.5%) of the collected *An. gambiae* complex species were identified as either *An. gambiae* or *An. coluzzii* (see the “Methods” section). Simulations from the posterior of the number of mosquitoes of each species in each village and month (see the “Methods” section) reflect the high heterogeneity in species composition between the four villages (Fig. 3). Bana and Bana Market showed consistently higher numbers of *An. coluzzii* relative to *An. gambiae* throughout the period (Fig. 3). In Pala there was typically more *An. gambiae* than *An. coluzzii,* although there is overlap in the posterior distributions of the monthly counts between the two species. In Souroukoudingan, estimated proportions of the two species are more similar, with higher numbers of *An. coluzzii* than *An. gambiae* on average.

### Statistical power to detect vector population suppression

We simulated CRCT data sets for trials lasting two years preceded by 1 year of baseline data collection by drawing counts of collected mosquitoes per house, month and cluster from the posterior distribution of the fitted geostatistical model (see the “Methods” section; Eqs. 1 and 2). We simulated counts of all mosquitoes that were morphologically identified as belonging to the *An. gambiae* complex, and counts disaggregated by species for the two predominant species, *An. gambiae* and *An. coluzzii* (see the “Methods” section; Eq. 5)*.*

We modelled two types of CRCT design, including parallel and step wedge designs (see the “Methods” section), and estimated the statistical power to detect a proportional reduction in mosquito counts, *G*, in the intervention compared to the control clusters. We considered three suppression effects, setting *G* to 50%, 70% and 90%. To represent mosquito release interventions targeting multiple species or only a single species, we compared cases where suppression acted on all *An. gambiae* complex species (including *An. gambiae*, *An. coluzzii* and *An. arabiensis*)*, An. gambiae* only, or *An*. *coluzzii* only. Our statistical analyses adjusted for baseline counts of the target vector species (see the “Methods” section). We note that our estimates of statistical power are stochastic, and our estimates of minimum trial sizes required to achieve a given statistical power are not exact.

**Fig. 4:**
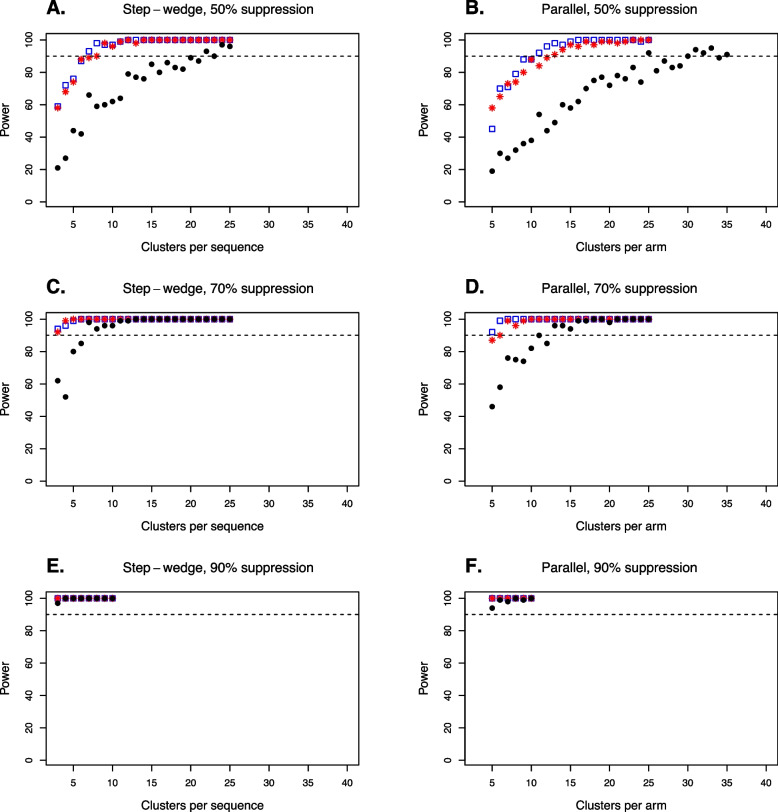
The power to detect a suppression effect *G* acting on targeted vector species. Blue square markers show the power when all *An. gambiae* complex species (*An. gambiae*, *An. coluzzii* and *An. arabiensis*) experience the suppression effect. Red asterisks and black circles show power values when only *An. coluzzii* (red markers) or only *An. gambiae* (black markers) are suppressed. Dotted lines show the 90% power threshold. Suppression effects of 50% (row **A**–**B**), 70% (row **C**–**D**) and 90% (row **E**–**F**) are shown. Both step wedge designs (column **A**, **C**, **E**) and parallel designs (column **B**, **D**, **F**) were modelled

For the smaller suppression effects (*G* = 50% and *G* = 70%), power depended on which vector species were targeted by the suppression effect (Fig. 4). Power was lowest when suppression acted on *An. gambiae* only (Fig. 4A–D). Power was similar when suppression acted on all three vector species compared to when suppression acted on *An. coluzzii* only, noting that targeting only *An. coluzzii* resulted in small decreases in power for the smallest suppression effect (*G* = 50%). For a suppression effect of 50% acting on all vector species, 90% power was achieved for 7 clusters per sequence (*C*_*s*_) for the step wedge trial design and 11 clusters per arm (*C*_*A*_) for the parallel trial design. When this suppression effect acted on *An. coluzzii* only, the step wedge design required 8 clusters per sequence to achieve 90% power and the parallel design required 13 clusters per arm. When suppression acted only on *An. gambiae*, the step wedge design required 22 clusters per sequence to achieve 90% power and the parallel design required 30 clusters per arm. Overall, the step wedge and parallel trial designs showed very similar efficiency, noting that the step wedge trial design has three sequences of *C*_*s*_ clusters per sequence and the parallel design has two arms of *C*_*A*_ clusters per arm (see the “Methods” section).

The larger trial sizes required when only *An. gambiae* experienced suppression can be explained by the relatively low numbers of *An. gambiae* collected, particularly in Bana centre and Bana market, where no *An. gambiae* were collected in a high proportion of houses (Additional file 1: Fig. S4). The relatively high proportion of zero counts for this species makes suppression effects more difficult to detect.

For stronger suppression effects, smaller trial sizes were required for 90% power, and there was less difference in required trial sizes depending on which vector species were targeted (Fig. 4). When suppression of *G* = 90% occurred, 90% power was achieved for the minimum trial size considered (*C*_*s*_ = 3 or *C*_*A*_ = 5), regardless of which vector species experienced the suppression effect (Fig. 4E, F).

### Effects of alternative monitoring strategies on statistical power

We explored the effects of alternative monitoring strategies on required trial sizes, including restricting PSC sample collection to the rainy season months (May–October) and varying the number of houses sampled in each monthly collection (see the “Methods” section).

#### Sampling during the rainy season only

**Fig. 5:**
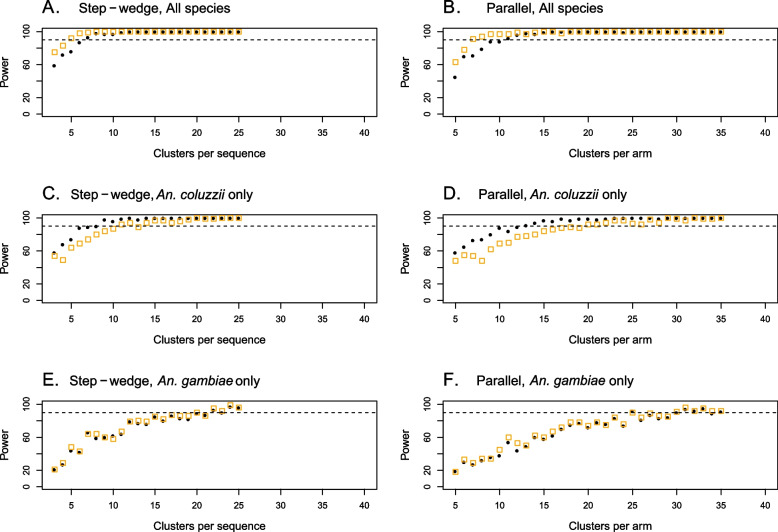
The effect sampling only during the rainy season (May–October) on the power to detect a suppression effect *G* acting on all *An. gambiae* complex species (*An. gambiae*, *An. coluzzii* and *An. arabiensis*). Power values when collections were made in all months of the year (black circles) and only the rainy season months (yellow squares) are shown. Results for suppression effects of *G* = 50% are shown. Dotted lines show the 90% power threshold. Rows show results when suppression affects all vector species (**A**, **B**), *An. coluzzii* only (**C**, **D**) and *An. gambiae* only (**E**, **F**). Both step wedge (column **A**, **C**, **E**) and parallel (column **B**, **D**, **F**) designs were modelled

Restricting PSC collections to the rainy season had nuanced effects on statistical power. When the lowest suppression effect (*G* = 50%) was experienced by all *An. gambiae* complex species, power increased when sampling was restricted to the rainy season for both step wedge and parallel designs (Fig. 5A, B). For the step wedge design, the required trial size for 90% power reduced from 7 to 5 clusters per sequence, and for the parallel design, the required trial size reduced from 11 to 7 clusters per arm. Mosquito counts in the rainy season are much higher than in the dry season (Fig. 1), so including dry season counts in the effect size estimation can increase the intercluster variation, despite the higher cluster sample sizes.

However, if the suppression effect targeted *An. coluzzii* only, restricting sampling to the rainy season reduced power (Fig. 5C, D). For the step wedge design, the required trial size for 90% power increased from 7 to 11 clusters per sequence, and for the parallel design, the required trial size increased from 13 to 20 clusters per arm. Restricting sampling to rainy season months did not affect power when the suppression effect targeted only *An. gambiae* (Fig. 5E, F). Thus, restricting sampling to the rainy season was only detrimental when *An. coluzzii* was the sole target of the suppression effect. *An. coluzzii* was present in the dry season months, with substantially higher dry season counts than *An. gambiae* in Bana and Bana market, and dry season sampling improved power to detect suppression. This could be because there were several houses with missing genotypic data (Additional file 1: Fig. S3), which reduced the cluster sample sizes for species-specific counts. Rainy season-only sampling further reduced sample sizes to the detriment of statistical power to detect suppression when *An. coluzzii* was the target species. This effect was not seen when all species experienced suppression because there were larger cluster sample sizes and less missing data on PSC counts of all *An. gambiae* complex species.

When the suppression effect was *G* = 70%, rainy season-only sampling had much smaller impacts on power (Additional file 1: Fig. S10). Reductions in power again occurred when *An. coluzzii* was the only species targeted, with negligible impacts when either *An. gambiae* or all three vector species experienced suppression. A suppression effect of *G* = 90% was sufficiently strong that restricting PSC collections to the rainy season months had no impact on statistical power, regardless of whether all species or only a single species experienced suppression (Additional file 1: Fig. S11).

#### Varying the number of houses sampled

**Fig. 6:**
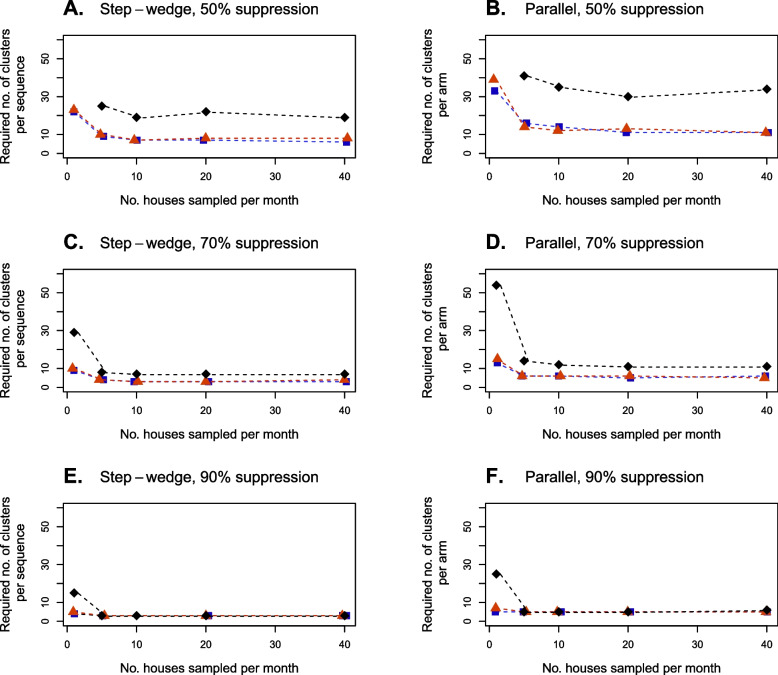
The effect of varying the number of houses sampled in each cluster per month on the number of clusters required to attain 90% power to detect a suppression effect *G.* Required cluster numbers when all *An. gambiae* complex species (blue lines and markers) experienced suppression, only *An. coluzzii* (red lines and markers) and only *An. gambiae* (black lines and markers) experienced suppression are shown. Results for suppression effects of *G* = 50% (**A**, **B**), *G* = 70% (**C**,**D**) and *G* = 90% (**E**, **F**) are shown. Both step-wedge (column **A**, **C**, **E**) and parallel (column **B**, **D**, **F**) designs were modelled. When only *An. gambiae* experienced the lowest suppression effect (*G* = 50%), sampling only one house per cluster was insufficient to attain 90% power for all trial sizes considered (results not shown). Mosquito collections are assumed to occur in all months of the year

The number of clusters required to provide 90% power to detect vector population suppression plateaued as the number of houses sampled in each cluster per month increased (Fig. 6). For the lowest suppression effect (*G* = 50%), using the step wedge design, required trial sizes plateaued when ten or more houses per month were sampled (Fig. 6A). Using the parallel design, required trial sizes also plateaued at ten houses per month, except when *An. gambiae* was the only vector species targeted by the suppression effect—in this case, required trial sizes plateaued when twenty or more houses per month were sampled (Fig. 6B). For the parallel design, more houses needed to be sampled to detect suppression acting on *An. gambiae* because PSC collections of *An. gambiae* contained fewer mosquitoes (Fig. 3). Under the step wedge design, however, required trial sizes plateaued at ten houses per month when *An. gambiae* was the only species targeted. This suggests that, for the step wedge design, statistical power is more robust to reductions in cluster sample sizes compared to the parallel design.

For a higher suppression effect of *G* = 70%, required trial sizes plateaued when five or more houses per month were sampled, for both parallel and step wedge designs, regardless of which species were considered as targets. When *G* was increased to 90%, required trial sizes plateaued when only one house per month was sampled when either all species were targeted by the suppression effect or when only *An. coluzzii* was targeted. However, when only *An. gambiae* was targeted, required trial sizes plateaued when five or more houses per month were sampled.

## Discussion

We developed statistical models to simulate data sets generated by CRCTs of interventions that suppress densities of adult malaria vectors, aiming to identify trial designs that achieve high statistical power with minimal sampling effort. Our models captured patterns of spatiotemporal variability observed in a five-year time series of malaria vector density measurements obtained in rural Burkina Faso, and thus provide empirically based estimates of statistical power. Statistical power depended on which vector species (within the *An. gambiae* complex) experienced the suppression effect of the intervention. Of the three vector species identified, *An. coluzzii* was most abundant in the PSC collections, and power was similar when suppression acted only on *An. coluzzii* to when suppression acted on all three vector species, provided the sampling protocol followed that used in the PSC collections [36]. When *An. gambiae* was the sole target of the suppression effect, power was substantially lower. Moreover, when *An. coluzzii* was the only species experiencing suppression, power was less robust to reducing sampling effort by restricting PSC collections to rainy season months. This was likely due to the substantial number of houses with missing data for genotyped mosquito samples, highlighting the additional challenges and resource requirements associated with monitoring densities of specific species within the *An. gambiae* complex.

There is potential for mosquito release strategies for population suppression to be developed in multiple malaria vector species. For example, gene drives that spread genes causing female infertility by targeting a female-specific exon in the *doublesex* gene are currently being developed in *An. gambiae* and *An. coluzzii* [17, 41, 42]*.* Strategies involving releasing sterile males without the onwards spread of modified genes through to the next generation, known as the sterile insect technique (SIT), have been investigated in *An. gambiae* [43], *An. coluzzii* [44] and *An. arabiensis* [45]. Outstanding challenges include developing efficient sex-separation methods in mass-reared mosquito colonies prior to male releases [42, 45, 46] and rearing males that will effectively compete with wild counterparts in mating [44]. Importantly, the ability of mosquito release interventions to target multiple malaria vector species will be critical to their efficacy in reducing human malaria cases [29], which is a strong motivation for overcoming the associated technical and logistical challenges.

We also obtained estimates of trial size requirements for a range of mosquito collection sampling regimes, in terms of the number of houses sampled from each village, and the times of year in which sampling occurs. Statistical power plateaued as the number of houses sampled per village increased, and we found that more houses were sampled in the PSC data set than were needed for approximately equivalent statistical power to detect vector suppression across the range considered, with 1–10 houses per village being sufficient in almost all cases. This small requirement on cluster sample sizes indicates that the precision of the estimated intervention effect is primarily determined by variation in mosquito counts between, rather than within, clusters. Our analyses considered trials with small numbers of clusters (typically < 60 clusters), and thus, we used simple analyses for estimating the intervention effect that did not consider sources of within-cluster variability in mosquito counts across houses [19]. Our geostatistical models indicated variable spatial distributions of mosquito counts within villages between months, with a lack of temporal consistency in the locations showing high mosquito numbers. This suggests that the inclusion of house-level variables may not contribute greatly to the explanatory power of the analyses, even if more detailed models were used. Interestingly, we found a negative temporal autocorrelation in the mosquito counts per house, which may possibly be due to a sustained effect of the PSC activity on mosquito densities in the house.

Our statistical model for estimating intervention effects adjusted for baseline data on counts of the target vector species by including a year of baseline data collection in our simulated data sets. Adjusting for baseline covariates that are predictive of the outcome of interest is recommended when there is variation between clusters with respect to these covariates [19, 47]. In the PSC data, mosquito counts and vector species composition were strongly variable across study sites. When we repeated our analyses without adjusting for baseline counts, power was greatly reduced (Additional file 1: Fig. S12). Thus, our results advocate for collecting baseline data in trials to assess suppression effects in species from the *An. gambiae* complex. In real trials, restricted randomisation approaches could alternatively be used to reduce imbalance across clusters [19]. Our statistical models representing variability in collections of *An. gambiae* complex mosquitoes are based on spatiotemporal data from four villages, limiting their ability to describe variability across a larger set of locations. We note, however, that collections took place over a five-year time period, so some variability due to changing conditions is captured. Nonetheless, we did not attempt to model restricted randomisation due to the limited number of clusters sampled in the PSC collections. In designing real trials, we recommend conducting baseline monitoring of vector densities over the full set of sites participating in the trial to assess whether intercluster variability deviates from the values observed in our data. Models could then be refitted to obtain updated estimates of trial design requirements.

We found that the parallel and step wedge designs were similarly efficient in terms of required numbers of clusters. Other studies have compared the efficiency of step wedge and parallel designs and found that either design can sometimes be more efficient, depending on the number of clusters, cluster sample sizes, and the level of intercluster correlation [48, 49]. For step wedge trials with larger trial sizes, analyses can gain power from longitudinal (before-and-after) comparisons within clusters [50], which are not taken into account by within-period analyses, which are recommended for small trial sizes [51–53]. Here we have used within-period analyses with adjustment for cluster-level mosquito counts at baseline. We note that analyses of step wedge designs can be biased if any secular temporal trends in the outcome measurements are not accurately accounted for [52, 53], and our simulation study did not incorporate or consider impacts of secular trends.

Our analyses considered only the female mosquitoes that were collected by PSC, but males were also collected and could potentially inform estimation of suppression effects. We did not include the counts of male mosquitoes because males have a lower propensity to rest indoors than females [54] and PSC collections may be less representative of true male densities. Power could be potentially increased by developing independent geostatistical models for the male and female counts and simulating CRCTs representing suppression impacts on both sexes, which could then be analysed jointly. Female malaria vectors do bite humans outdoors, and an increase in outdoor biting propensity has been observed in some areas following widespread use of indoor insecticidal interventions [55]. PSC methods capture only indoor resting mosquitoes and do not represent outdoor biting vector populations, which is a limitation of using PSC for measuring mosquito densities.

Our assumption of a temporally constant suppression effect is a simplification of the impacts of mosquito release interventions, which will inevitably cause temporally varying suppression effects. We have assumed a Poisson model of mosquito counts, whereby the suppression intervention linearly reduces the variance in mosquito counts. The nature of the variability associated with the suppression effect will be specific to the intervention and the intensity of the releases. Extensions of our analyses to incorporate time-varying suppression effects will be important to consider and could be informed by predictions of mechanistic models of vector population dynamics (e.g. [56, 57]). We note, however, that we model CRCTs where the endpoint is average suppression across a two-year time period, rather than temporal variation in suppression throughout the trial. Thus, our results are a parsimonious representation of vector suppression and constitute a necessary first step.

Our analysis also did not consider potential spillover of the intervention into control clusters [58], which could occur with mosquito release interventions that introduce spatially spreading modifications, such as gene drives. Spillover effects could be mitigated by geographic separation of clusters to allow for buffer zones [19], although there are challenges involved in estimating the required size of the buffer zones a priori. Alternatively, a CRCT of *Wolbachia* releases in *Ae. aegypti* in Yogyakarta, Indonesia, allowed for spillover based on calculations showing minimal effects on statistical power [59]. Further modelling research to estimate the impacts of spillover on trial design requirements will be needed to inform first field trials of spatially spreading interventions such as novel low-threshold gene drives.

When released modified genes persist in wild mosquito populations for many generations, as is anticipated with low-threshold gene drives, they could potentially introgress into closely related species through hybridisation. This could occur between the sibling species *An. coluzzii* and *An. gambiae*, which are known to hybridise in the wild [60]. It is uncertain whether this would occur within the duration of a CRCT, and it will be important for trials of gene drive releases to include monitoring to detect the modified gene in closely related species that could potentially hybridise with the released species [18].

## Conclusions

Our modelling analyses characterise CRCTs for detecting malaria vector population suppression by estimating trial size requirements for a range of trial designs, vector species targets, suppression efficacies and sampling strategies. Several novel mosquito release interventions for malaria control are currently progressing towards field applications, but their efficacy in vector population suppression has not yet been rigorously assessed using CRCT methods. CRCT approaches will likely be important tools in developing a robust understanding of suppression efficacy and optimising release strategies. Encouragingly, our results indicate that large CRCTs are not necessary to provide statistically powered estimates of moderate to high suppression effects.

## Methods

### Time series of mosquito density measurements

From July 2012 to July 2014 and from January 2017 to December 2019, indoor resting mosquitoes were collected each month from housing compounds in four villages near Bobo Dioulasso: Bana centre, Bana market, Pala and Souroukoudingan [36, 61]. These villages are all within 30 km of Bobo Dioulasso, with Bana Market being a peripheral district of Bana that is separated from Bana Center by a semi-permanent river [36]. A high coverage of ITNs in the region has been reported in recent years [36]. Mosquitoes were collected using pyrethrum spray catches (PSC); details of the mosquito collection procedures are provided in Epopa et al. [36]. In summary, in Bana, Pala and Souroukoudingan, twenty houses in each village were sprayed per month, while in the smaller settlement of Bana Market six houses were sprayed per month. For each house, the spray date and GPS (Global Positioning System) positions were recorded. Of the twenty houses, ten were selected at random each month from each village, and a fixed set of ten houses in each village were repeatedly sampled each month. In Bana Market, all sampled houses were within a fixed set of houses that were repeatedly sampled each month. The fixed houses were spread to represent the geography and extent of each village. For some months, there are some missing records, and count data for less than twenty houses is available, or less than six houses in the case of Bana Market (Additional file 1: Fig. S2).

In this study we use data on counts of collected mosquito species from the *An. gambiae* complex, which were identified morphologically. From January 2017 onwards, a subset of ninety mosquitoes identified as members of the *An. gambiae* complex were retained from each village’s monthly collection for species identification by Polymerase Chain Reaction (PCR) [62]. The retained samples were selected at random from the total catch in each house, where the number retained per house was proportional to the total catch in each house. This ensured that the samples of specimens retained for species identification were representative of the full data set at the house level. For some months, species identification failed for some of the collected mosquitoes, so the data set contains missing information on species for some samples (Additional file 1: Fig. S3). Of the species identified from the *An. gambiae* complex, the vast majority were either *An. gambiae* or *An. coluzzii*, with *An. arabiensis* making up 6.5% of all samples identified. Vector species composition was found to differ markedly across the four villages. In Bana and Bana market, about 93% of all collected mosquitoes from the *An. gambiae* complex were identified as *An. coluzzii.* In Souroukoudingan, 57% were *An. coluzzii* and in Pala 19% were *An. coluzzii* [36].

### Geostatistical models of mosquito counts

We fitted a Bayesian spatiotemporal geostatistical model to the counts of adult female mosquitoes in each house to estimate the patterns of variability in the counts across houses, villages and months of the year, accounting for spatiotemporal autocorrelation in the count data. We modelled the counts of only female, and not male, mosquitoes, because PSC data is likely to better represent female vector densities, as males have a lower propensity to rest indoors [54] (see the “Discussion” section). We model the number of female mosquitoes from the *An. gambiae* complex collected in each house, *h*, month, *m,* and village, *v*, denoted ${y}_{hmv}$, as a Poisson distribution:

1 $$y_{hmv}\sim Pois\left(\lambda_{hmv}\left|f\left(x_{h,}m\right),\right.\theta\right)$$

The linear predictor is modelled using a Gaussian process regression formulation:

2 $$\log\left(\lambda_{hmv}\right)=f\left(x_{h,}m\right)+\mu_v+v_h$$

where ${{\varvec{x}}}_{h}$ is the location of house *h* (in World Geodetic System (WGS)) 84 latitude and longitude coordinates), $f\left({{\varvec{x}}}_{h},m\right)$ is a multivariate Gaussian process modelled by a spatiotemporal Gaussian Markov Random Field (GMRF), and ${\mu }_{v}$ and ${\nu }_{h}$ are independently distributed Gaussian random effects representing overdispersion at the village and house levels, respectively. We define a Bayesian hierarchical formulation for the model using a vector of prior probability distributions for the hyperparameters $\theta =[{\varvec{\psi}},{\sigma }_{v},{\sigma }_{h}]$, where ${\varvec{\psi}}$ are the parameters of $f\left({{\varvec{x}}}_{h},m\right)$ (see Additional file 1), and ${\sigma }_{v}$ and ${\sigma }_{h}$ are the standard deviations of the Gaussian random effects ${\mu }_{v}$ and ${\nu }_{h}$. Posterior distributions of $f\left({{\varvec{x}}}_{h},m\right)$, ${\mu }_{v}$ and ${\nu }_{h}$ were then estimated by fitting the model using the R-INLA package [39], which approximates $f\left({{\varvec{x}}}_{h},m\right)$ using a stochastic partial differential equation (SPDE) approach [63]. This involves solving for $f\left({{\varvec{x}}}_{h},m\right)$ at a discrete set of points which are the nodes of a mesh constructed using a Delauney triangulation. We constructed a mesh covering an area encompassing all four villages, with a fine resolution within the area of each village and a coarse resolution elsewhere (Additional file 1: Fig. S1). Code for implementing the geostatistical models developed in our study using R-INLA is available on GitHub [64].

### Simulating field trials to detect mosquito population suppression

We simulate data sets representing CRCTs aiming to detect suppression of a targeted mosquito vector species resulting from a vector control intervention, assuming a constant suppression effect *G* throughout the trial period. We considered two approaches to CRCT design, including a parallel and a step wedge design. While parallel CRCTs are the standard approach used in trials of vector control interventions, step wedge designs allow the times of intervention implementation in each cluster to be staggered, such that some clusters receive the intervention at later times than others. This may be preferable for novel strategies involving releasing GM mosquitoes and gene drives, which have not yet been tested in the field.

For both approaches, we simulated data for a set of treatment and control clusters, assuming that a cluster is represented by a village, and that mosquito count data are collected from each cluster following the same protocol as that used in the PSC collections for the four study villages. Thus, the number of houses sampled in each cluster, month and year is assumed to be equal to that for which PSC data was obtained (below we describe a modification where the number of houses per cluster is varied). For each cluster, we simulated the number of female mosquitoes collected from each house *h* and month* m* by drawing from the posterior distributions of $f\left({{\varvec{x}}}_{h},m\right)$, ${\mu }_{v}$ and ${\nu }_{h}$ obtained from the fitted spatiotemporal model. The simulated counts ${\widetilde{y}}_{hmv}^{s}$ were then given by drawing from a Poisson distribution with a mean of

3 $$\widetilde\lambda_{hmv}^s=exp\left(\widetilde f^s\left(x_h,m\right)+\widetilde\mu_v^s+\widetilde v_h^s\right)$$

where ${\widetilde{f}}^{s}\left({{\varvec{x}}}_{h},m\right)$ is the *s*th random draw from the posterior distribution of$f\left({{\varvec{x}}}_{h},m\right)$, and similarly ${\widetilde{\mu }}_{v}^{s}$, and ${\widetilde{\nu }}_{h}^{s}$ are random draws from the fitted posterior distributions of these random effects. We simulate a number of data sets, *S,* denoting *s* = 1,…,*S* as the simulation index. Code and vignettes for generated the simulated data sets is available on Github [64].

#### Simulating parallel CRCT designs

We simulated data sets for parallel designs where clusters are randomly allocated to receive the intervention or to act as controls, with all the intervention clusters receiving the intervention simultaneously. We assume a balanced design with equal numbers of clusters per arm, *C*_*A*_. We consider trials lasting two years, where the simulated data for each year is obtained by selecting random posterior draws of counts ${\widetilde{y}}_{hmv}^{s}$ for all months in each year (we also consider cases where only rainy season months are sampled, see below). We assume here that the intervention targets all *An. gambiae* complex species recorded in the PSC collections (simulations assuming only a single vector species is targeted are considered below). Thus, for treatment clusters, we multiply ${\widetilde{y}}_{hmv}^{s}$ by a factor $1-G,$ where *G* is the assumed level of suppression of the target vector population. We also simulate a year of pre-trial baseline data collection, whereby mosquito counts ${\widetilde{y}}_{hmv}^{s}$ are drawn for all clusters for all months in a single year, and no clusters experience the suppression effect. We note that the locations of the houses for which counts ${\widetilde{y}}_{hmv}^{s}$ are simulated vary across years following the sampling patterns in the PSC data set, which had missing records for some houses in certain months and years (see Additional file 1).

For each simulated data set *s*, we then obtain an estimate of the suppression effect *G* by performing a linear regression on cluster-level summaries of the logarithm of the mean number of mosquitoes collected per house in each cluster, where the log transform is applied to reduce skewness in the cluster-level counts [19]. We based our inference on cluster-level summaries because our field trial simulations consider small numbers of clusters [19]. We used the following regression model to estimate the suppression effect *G*, adjusting for the total cluster-level mosquito counts at baseline:

4 $$\log\left(\overline Y_v^s\right)=\log\left(1-\widehat G^s\right)\;I^s+\alpha\log\left(\overline Y_{B,v}^s\right)$$

where, for simulated data set *s*, ${\overline{Y} }_{v}^{s}$ is the mean number of mosquitoes per house collected from village *v* over the course of the two-year trial period, ${I}^{s}$ is a binary variable indicated whether or not the village received the intervention throughout the trial, ${\overline{Y} }_{B,v}^{s}$ is mean number of mosquitoes per house collected from village *v* during the one-year baseline collection period and $\alpha$ is a constant coefficient. We included adjustment for baseline mosquito counts as these were heterogeneous across the villages, with an intercluster coefficient of variation (typically denoted *k* [19]) in the mean observed mosquito counts ${y}_{hmv}$ per house across all months and years of *k* = 0.37. This led to imbalances in the primary outcome measure ${\overline{Y} }_{v}^{s}$ between treatment arms, which we mitigated by adjusting for cluster level mosquito counts at baseline [19].

For each value of *C*_*A*_, we estimated the statistical power to detect the suppression impact *G* by estimating ${\widehat{G}}^{s}$ for *S* = 100 simulated data sets and calculating the proportion of estimates that were significant based on a two-tailed *p*-value of less than 0.05 [65]. Inference was performed in Stata [66] [64].

#### Simulating step wedge CRCT designs

We simulated step wedge trial designs whereby clusters are divided into sequences and the time at which the intervention is implemented varies between sequences (Additional file 1: Fig. S5). Interventions are timed to begin at the start of fixed time periods; here we assume that each period lasts 1 year. We chose a design with three sequences and two time periods, with the clusters in the first sequence receiving the intervention in both periods, and the clusters in the second sequence receiving the intervention in the second period only. Clusters in the third sequence do not receive the intervention and serve as controls throughout the trial. As above, we simulated a year of baseline mosquito sampling for all clusters, so each simulated data set covered a three-year period.

We simulated data sets for step wedge designs varying the number of clusters per sequence, *C*_*S*_, where the simulated data for each cluster and sequence was obtained by selecting random posterior draws of the counts ${\widetilde{y}}_{hmv}^{s}$ for all houses and months in the three-year period. We multiply ${\widetilde{y}}_{hmv}^{s}$ in the clusters receiving the intervention by the suppression effect 1-*G* as described above. We simulated 100 data sets for each value of *C*_*S*_. Due to the small numbers of clusters that we consider in our simulated trials, we estimate ${\widehat{G}}^{s}$ for each data set using a within-period analysis [51], again adjusting for the baseline mosquito counts in each cluster. We estimate the intervention effect using the same linear regression model as described above for the parallel trial design (Eq. 4). Confidence intervals for ${\widehat{G}}^{s}$ were estimated using a permutation test [51] with 500 permutations. Statistical power for each value of *C*_*S*_ was calculated as the proportion (out of *S* = 100) of estimates that were statistically significant based on a p-value of 0.05.

#### Simulating suppression on a single vector species

Species identification was performed on mosquitoes collected by PSC for samples collected from January 2017 to December 2019, as discussed above. For these years, we simulate the number of female *An. coluzzii* collected in each house, month and village, ${\widetilde{c}}_{hmv}^{s}$, by:

5 $$\widetilde c_{hmv}^s=\widetilde y_{hmv}^s\;\widetilde Z^s/S_{hmv}$$

Here, ${S}_{hmv}$ is number of female mosquitoes in the subset that were retained for species identification in house *h*, month *m* and village *v.* ${\widetilde{Z}}^{s}$ is a random variable sampled from a binomial distribution with a number of trials ${S}_{hmv}$ and a mean probability equal to the proportion of ${S}_{hmv}$ that were identified as *An. coluzzii* (denoted ${p}_{hmv}^{c}$) by genotyping*.* The number of female *An. gambiae* collected in each house, month and village,${\widetilde{g}}_{hmv}^{s}$, was simulated in the same way. We note that ${S}_{hmv}$ and the species composition of these samples varies across years in the PSC data, and our simulated counts ${\widetilde{c}}_{hmv}^{s}$ and ${\widetilde{g}}_{hmv}^{s}$ incorporate this yearly variation (see Additional file 1 for further details).

We simulated data sets for step wedge and parallel CRCT designs in a similar way to the above methodology for simulating counts across all species (${\widetilde{y}}_{hmv}^{s}$). For each cluster, the simulated data for each year is obtained by selecting random posterior draws to calculate the counts ${\widetilde{y}}_{hmv}^{s}$ for all months. Corresponding values of ${\widetilde{c}}_{hmv}^{s}$ and ${\widetilde{g}}_{hmv}^{s}$ were then calculated using Eq. 5. For parallel designs we simulated *S* = 100 data sets for each value of the number of clusters per arm, *C*_*A*_; for step wedge designs we did the same for each value the number of clusters per sequence, *C*_*S*_. In the intervention clusters, values of ${\widetilde{c}}_{hmv}^{s}$ and ${\widetilde{g}}_{hmv}^{s}$ were multiplied by the suppression effect 1-*G.* For each data set we estimated ${\widehat{G}}^{s}$ using linear regression models similar to those described above for parallel and step wedge designs (Eq. 4), modified to represent the counts of only the target vector species:

6 $$\text{log}\left({\overline{C} }_{v}^{s}\right)=\text{log}(1-{\widehat{G}}^{s}){I}^{s}+\alpha {\text{log}(\overline{C} }_{B,v}^{s})$$

where ${\overline{C} }_{v}^{s}$ is the mean counts per house of *An. coluzzii* in village *v* over the trial period and ${\overline{C} }_{v,B}^{s}$ is the mean counts per house over the baseline period. The same approach was used to model suppression effects acting only on *An. gambiae*. Statistical power was again calculated as the proportion of the *S* = 100 estimates that were significant based on a two-tailed *p*-value of less than 0.05.

#### Simulating alternative sampling strategies

An important aspect of CRCT design is selecting sampling strategies with sample sizes that are sufficient to achieve the required statistical power but not unnecessarily large, leading to wasted resources. We simulated CRCT data sets for a range of alternative monitoring strategies. We first restricted the period of PSC collections to the rainy season months of May–October, which is the period when the majority of the mosquitoes are collected, by using only the data for each cluster, ${\widetilde{y}}_{hmv}^{s}$, for months *m* = May–October inclusive. Second, we varied the number of houses from which monthly PSC collections were made in each cluster. We simulated data sets for different sampling protocols where the number of houses sampled in each village and month, *H*, was set to either *H* = 1, 5, 10, 20 or 40 houses, noting that this sample size could not always be attained for some clusters due to missing data. Our methodology for generating these simulated data sets is as follows. If the value of *H* was less than the number of houses sampled per village and month by the PSC collections, a subset of *H* houses was randomly sampled without replacement from the full set of houses from which mosquitoes were collected. If *H* was greater than the number of houses sampled by the PSC collections, house locations were sampled twice. We note that, for Bana, Pala and Souroukoudingan, twenty houses were sampled by PSC each month, whereas for Bana Market, only six houses were sampled by PSC per month. Thus, for clusters where data was drawn from the Bana Market houses, the maximum number of houses sampled was *H* = 12.

## Supplementary Information

Additional file 1: Fig. S1. The Delaunay triangulation mesh used in the SPDE model. Fig. S2. The number of compounds from which PSC collections were made per month across all months in Bana village, Bana market, Pala and Souroukoudingan. Fig. S3. The number of female mosquitoes genotyped for species identification in each village and month across all months in the period 2017-2019. Fig. S40. The distributions of the numbers of mosquitoes of each species collected per compound per month from each village. Fig. S5. The design of the step-wedge trial modelled in this study. Clusters shaded in blue receive the intervention and unshaded clusters do not receive the intervention. Fig. S6-S8. Posterior predicted mean counts of all *An. gambiae *complex species at each mesh node of the SPDE model across Bana village, Pala and Souroukoudingan. Fig. S9. Posterior predicted mean counts of all *An. gambiae *complex species for each month at ten randomly selected locations in Bana village, Pala, and Souroukoudingan. Fig. S10-S11. The effect sampling only during the rainy season (May-October) on the power to detect a suppression effect *G*=70% and 90%acting on all *An. gambiae *complex species (*An. gambiae*, *An. coluzzii*, and *An. arabiensis*). Fig. S12. The power to detect a suppression effect *G *acting on targeted vector species when no baseline data on mosquito counts is available.

## Data Availability

Data for generating the simulated CRCT data sets analysed in this study is provided on GitHub (64). Computer code in R and Stata and vignettes for reproducing the statistical modelling analyses developed in this study is provided on Github (64).

## Abbreviations

CI: Credible interval
CRCT: Cluster randomised control trial
GPS: Geographic Positioning System
GM: Genetically modified
ITN: Insecticide-treated net
IRS: Indoor residual spraying
PCR: Polymerase chain reaction
PSC: Pyrethrum spray catch
SIT: Sterile insect technique
SPDE: Stochastic partial differential equation
WGS: World Geodetic System
WHO: World Health Organisation

## References

[1] Weiss DJ, Lucas TCD, Nguyen M, Nandi AK, Bisanzio D, Battle KE, et al. Mapping the global prevalence, incidence, and mortality of *Plasmodium falciparum*, 2000–17: a spatial and temporal modelling study. Lancet. 2019;394(10195):322–31. 31229234 10.1016/S0140-6736(19)31097-9 PMC6675740

[2] Ritchie SA, Devine GJ, Vazquez-Prokopec GM, Lenhardt AE, Manrique-Saide P, Scott TW. Insecticide-based approaches for dengue vector control. In: Spitzen J, Takken W, editors. Koenraadt JMC. Innovative strategies for vector control. Ecology and control of vector-borne diseases. Wageningen: Wageningen Academic Publishers; 2021. p. 59–89.

[3] Hancock PA, Ochomo E, Messenger LA. Genetic surveillance of insecticide resistance in African *Anopheles* populations to inform malaria vector control. Trends Parasitol. 2024;40(7):604–18. 38760258 10.1016/j.pt.2024.04.016

[4] World Health Organization. World Malaria Report 2024. Geneva: World Health Organization; 2024.

[5] Klassen W. Introduction: development of the sterile insect technique for African malaria vectors. Malar J. 2009;8(2):I1. 19917069 10.1186/1475-2875-8-S2-I1 PMC2777321

[6] Nolan T, Papathanos P, Windbichler N, Magnusson K, Benton J, Catteruccia F, et al. Developing transgenic Anopheles mosquitoes for the sterile insect technique. Genetica. 2011;139(1):33–9. 20821345 10.1007/s10709-010-9482-8

[7] Lees RS, Carvalho DO, Bouyer J. Potential Impact of Integrating the Sterile Insect Technique into the Fight against Disease-Transmitting Mosquitoes. In: Dyck VA, Hendrichs J, Robinson AS, editors. Sterile Insect Technique. 2nd ed: CRC Press; 2021.

[8] Dickens BL, Yang J, Cook AR, Carrasco LR. Time to empower release of insects carrying a dominant lethal and *Wolbachia* against Zika. Open Forum Infect Dis. 2016;3(2):ofw103. 27419175 10.1093/ofid/ofw103 PMC4943532

[9] Lim JT, Bansal S, Chong CS, Dickens B, Ng Y, Deng L, et al. Efficacy of *Wolbachia* mediated sterility to reduce the incidence of dengue: a synthetic control study in Singapore. Lancet Microbe. 2024;5(5):e422–32. 38342109 10.1016/S2666-5247(23)00397-X

[10] Carvalho DO, McKemey AR, Garziera L, Lacroix R, Donnelly CA, Alphey L, et al. Suppression of a field population of *Aedes aegypti* in Brazil by sustained release of transgenic male mosquitoes. PLoS Negl Trop Dis. 2015;9(7):e0003864. 26135160 10.1371/journal.pntd.0003864 PMC4489809

[11] Alphey LS, Crisanti A, Randazzo FF, Akbari OS. Opinion: Standardizing the definition of gene drive. Proc Natl Acad Sci U S A. 2020;117(49):30864–7. 33208534 10.1073/pnas.2020417117 PMC7733814

[12] Hammond AM, Galizi R. Gene drives to fight malaria: current state and future directions. Pathog Glob Health. 2017;111(8):412–23. 29457956 10.1080/20477724.2018.1438880 PMC6066861

[13] Marshall JM, Akbari OS. Can CRISPR-based gene drive be confined in the wild? A question for molecular and population biology. ACS Chem Biol. 2018;13(2):424–30. 29370514 10.1021/acschembio.7b00923

[14] Metzloff M, Yang E, Dhole S, Clark AG, Messer PW, Champer J. Experimental demonstration of tethered gene drive systems for confined population modification or suppression. BMC Biol. 2022;20(1):119. 35606745 10.1186/s12915-022-01292-5 PMC9128227

[15] Oxitec. Djibouti advances the fight against malaria with launch of first full pilot season of friendly™ mosquitoes in Africa, targeting the invasive *Anopheles stephensi*: https://www.oxitec.com/en/news/djiboutipilotseason; 2024 [

[16] Burt A. Site-specific selfish genes as tools for the control and genetic engineering of natural populations. Proc R Soc Lond B. 2003;270(1518):921–8. 10.1098/rspb.2002.2319 PMC1691325 12803906

[17] Hammond A, Pollegioni P, Persampieri T, North A, Minuz R, Trusso A, et al. Gene-drive suppression of mosquito populations in large cages as a bridge between lab and field. Nat Commun. 2021. 10.1038/s41467-021-24790-6. 34321476 10.1038/s41467-021-24790-6 PMC8319305

[18] Connolly JB, Burt A, Christophides G, Diabate A, Habtewold T, Hancock PA, et al. Considerations for first field trials of low-threshold gene drive for malaria vector control. Malar J. 2024;23(1):156. 38773487 10.1186/s12936-024-04952-9 PMC11110314

[19] Hayes RJ, Moulton LH. Cluster Randomised Trials. 2nd ed: CRC Press, Taylor and Francis Group; 2017.

[20] World Health Organization. Design of epidemiological trials for vector control products: report of a WHO Expert Advisory Group, Chateau de Penthes, Geneva Geneva: World Health Organization; 2017 [Available from: https://apps.who.int/iris/bitstream/handle/10665/255854/WHO-HTM-NTD-VEM-2017.04-eng.pdf.

[21] Accrombessi M, Cook J, Dangbenon E, Yovogan B, Akpovi H, Sovi A, et al. Efficacy of pyriproxyfen-pyrethroid long-lasting insecticidal nets (LLINs) and chlorfenapyr-pyrethroid LLINs compared with pyrethroid-only LLINs for malaria control in Benin: a cluster-randomised, superiority trial. Lancet. 2023;401(10375):435–46. 36706778 10.1016/S0140-6736(22)02319-4

[22] Maiteki-Sebuguzi C, Gonahasa S, Kamya MR, Katureebe A, Bagala I, Lynd A, et al. Effect of long-lasting insecticidal nets with and without piperonyl butoxide on malaria indicators in Uganda (LLINEUP): final results of a cluster-randomised trial embedded in a national distribution campaign. Lancet Infect Dis. 2023;23(2):247–58. 36174592 10.1016/S1473-3099(22)00469-8

[23] Mosha JF, Kulkarni MA, Lukole E, Matowo NS, Pitt C, Messenger LA, et al. Effectiveness and cost-effectiveness against malaria of three types of dual-active-ingredient long-lasting insecticidal nets (LLINs) compared with pyrethroid-only LLINs in Tanzania: a four-arm, cluster-randomised trial. Lancet. 2022;399(10331):1227–41. 35339225 10.1016/S0140-6736(21)02499-5 PMC8971961

[24] Protopopoff N, Mosha JF, Lukole E, Charlwood JD, Wright A, Mwalimu CD, et al. Effectiveness of a long-lasting piperonyl butoxide-treated insecticidal net and indoor residual spray interventions, separately and together, against malaria transmitted by pyrethroid-resistant mosquitoes: a cluster, randomised controlled, two-by-two factorial design trial. Lancet. 2018;391(10130):1577–88. 29655496 10.1016/S0140-6736(18)30427-6 PMC5910376

[25] Zheng X, Zhang D, Li Y, Yang C, Wu Y, Liang X, et al. Incompatible and sterile insect techniques combined eliminate mosquitoes. Nature. 2019;572(7767):56–61. 31316207 10.1038/s41586-019-1407-9

[26] Cannet A, Simon-Chane C, Akhoundi M, Histace A, Romain O, Souchaud M, et al. Deep learning and wing interferential patterns identify *Anopheles* species and discriminate amongst *Gambiae* complex species. Sci Rep. 2023;13(1):13895. 37626130 10.1038/s41598-023-41114-4 PMC10457333

[27] Connolly JB, Romeis J, Devos Y, Glandorf DCM, Turner G, Coulibaly MB. Gene drive in species complexes: defining target organisms. Trends Biotechnol. 2023;41(2):154–64. 35868886 10.1016/j.tibtech.2022.06.013

[28] Zhong D, Hemming-Schroeder E, Wang X, Kibret S, Zhou G, Atieli H, et al. Extensive new *Anopheles* cryptic species involved in human malaria transmission in western Kenya. Sci Rep. 2020;10(1):16139. 32999365 10.1038/s41598-020-73073-5 PMC7527330

[29] Hancock PA, North A, Leach AW, Winskill P, Ghani AC, Godfray HCJ, et al. The potential of gene drives in malaria vector species to control malaria in African environments. Nat Commun. 2024;15(1):8976. 39419965 10.1038/s41467-024-53065-z PMC11486997

[30] Staedke SG, Gonahasa S, Dorsey G, Kamya MR, Maiteki-Sebuguzi C, Lynd A, et al. Effect of long-lasting insecticidal nets with and without piperonyl butoxide on malaria indicators in Uganda (LLINEUP): a pragmatic, cluster-randomised trial embedded in a national LLIN distribution campaign. Lancet. 2020;395(10232):1292–303. 32305094 10.1016/S0140-6736(20)30214-2 PMC7181182

[31] Yukich J, Eisele TP, terKuile F, Ashton R, Staedke S, Harris AF, et al. Master statistical analysis plan: attractive targeted sugar bait phase III trials in Kenya, Mali, and Zambia. Trials. 2023;24(1):771. 38031086 10.1186/s13063-023-07762-7 PMC10685482

[32] Accrombessi M, Cook J, Dangbenon E, Yovogan B, Akpovi H, Sovi A, et al. Efficacy of pyriproxyfen-pyrethroid long-lasting insecticidal nets (LLINs) and chlorfenapyr-pyrethroid LLINs compared with pyrethroid-only LLINs for malaria control in Benin: a cluster-randomised, superiority trial. Lancet. 2023;401(10375):435–46. 36706778 10.1016/S0140-6736(22)02319-4

[33] Lehmann T, Dao A, Yaro AS, Diallo M, Timbiné S, Huestis DL, et al. Seasonal variation in spatial distributions of *Anopheles gambiae* in a Sahelian village: evidence for aestivation. J Med Entomol. 2014;51(1):27–38. 24605449 10.1603/me13094 PMC3960504

[34] Ndiath MO, Sarr J-B, Gaayeb L, Mazenot C, Sougoufara S, Konate L, et al. Low and seasonal malaria transmission in the middle Senegal River basin: identification and characteristics of Anopheles vectors. Parasit Vectors. 2012;5(1):21. 22269038 10.1186/1756-3305-5-21 PMC3274455

[35] Kosgei J, Gimnig JE, Moshi V, Omondi S, McDermott DP, Donnelly MJ, et al. Comparison of different trapping methods to collect malaria vectors indoors and outdoors in western Kenya. Malar J. 2024;23(1):81. 38493098 10.1186/s12936-024-04907-0 PMC10943837

[36] Epopa PS, Collins CM, North A, Millogo AA, Benedict MQ, Tripet F, et al. Seasonal malaria vector and transmission dynamics in western Burkina Faso. Malar J. 2019;18(1):113. 30940141 10.1186/s12936-019-2747-5 PMC6444393

[37] Cameletti M, Lindgren F, Simpson D, Rue H. Spatio-temporal modeling of particulate matter concentration through the SPDE approach. AStA Adv Stat Anal. 2013;97(2):109–31.

[38] Fuglstad G-A, Daniel S, Finn L, Rue H. Constructing priors that penalize the complexity of Gaussian random fields. J Am Stat Assoc. 2019;114(525):445–52.

[39] Lindgren F, Rue H, Lindstrom J. An explicit link between Gaussian fields and Gaussian Markov random fields: the stochastic partial differential equation approach. J R Stat Soc Ser B Stat Methodol. 2011;73:423–98.

[40] Simpson D, Rue H, Riebler A, Martins TG, Sørbye SH. Penalising model component complexity: a principled, practical approach to constructing priors. Stat Sci. 2017;32(1):1–28.

[41] Yao FA, Millogo A-A, Epopa PS, North A, Noulin F, Dao K, et al. Mark-release-recapture experiment in Burkina Faso demonstrates reduced fitness and dispersal of genetically-modified sterile malaria mosquitoes. Nat Commun. 2022;13(1):796. 35145082 10.1038/s41467-022-28419-0 PMC8831579

[42] Apte RA, Smidler AL, Pai JJ, Chow ML, Chen S, Mondal A, et al. Eliminating malaria vectors with precision-guided sterile males. Proc Natl Acad Sci U S A. 2024;121(27):e2312456121. 38917000 10.1073/pnas.2312456121 PMC11228498

[43] Klein TA, Windbichler N, Deredec A, Burt A, Benedict MQ. Infertility resulting from transgenic I-PpoI male *Anopheles gambiae* in large cage trials. Pathog Glob Health. 2012;106(1):20–31. 22595271 10.1179/2047773212Y.0000000003 PMC4001508

[44] Maïga H, Damiens D, Niang A, Sawadogo SP, Fatherhaman O, Lees RS, et al. Mating competitiveness of sterile male *Anopheles coluzzii* in large cages. Malar J. 2014;13:460. 25424008 10.1186/1475-2875-13-460 PMC4258930

[45] Mashatola T, Ndo C, Koekemoer LL, Dandalo LC, Wood OR, Malakoane L, et al. A review on the progress of sex-separation techniques for sterile insect technique applications against *Anopheles arabiensis*. Parasit Vectors. 2018;11(2):646. 30583746 10.1186/s13071-018-3219-4 PMC6304763

[46] Papathanos PA, Bourtzis K, Tripet F, Bossin H, Virginio JF, Capurro ML, et al. A perspective on the need and current status of efficient sex separation methods for mosquito genetic control. Parasit Vectors. 2018;11(2):654. 30583720 10.1186/s13071-018-3222-9 PMC6304774

[47] Nevins P, Davis-Plourde K, Pereira Macedo JA, Ouyang Y, Ryan M, Tong G, et al. A scoping review described diversity in methods of randomization and reporting of baseline balance in stepped-wedge cluster randomized trials. J Clin Epidemiol. 2023;157:134–45. 36931478 10.1016/j.jclinepi.2023.03.010 PMC10546924

[48] Hemming K, Girling A, Martin J, Bond SJ. Stepped wedge cluster randomized trials are efficient and provide a method of evaluation without which some interventions would not be evaluated. J Clin Epidemiol. 2013;66(9):1058–9. 23849737 10.1016/j.jclinepi.2012.12.020

[49] Kotz D, Spigt M, Arts ICW, Crutzen R, Viechtbauer W. The stepped wedge design does not inherently have more power than a cluster randomized controlled trial. J Clin Epidemiol. 2013;66(9):1059–60. 23953488 10.1016/j.jclinepi.2013.05.004

[50] Thompson JA, Hemming K, Forbes A, Fielding K, Hayes R. Comparison of small-sample standard-error corrections for generalised estimating equations in stepped wedge cluster randomised trials with a binary outcome: a simulation study. Stat Methods Med Res. 2021;30(2):425–39. 32970526 10.1177/0962280220958735 PMC8008420

[51] Thompson JA, Davey C, Fielding K, Hargreaves JR, Hayes RJ. Robust analysis of stepped wedge trials using cluster-level summaries within periods. Stat Med. 2018;37(16):2487–500. 29635789 10.1002/sim.7668 PMC6032886

[52] Thompson JA, Fielding KL, Davey C, Aiken AM, Hargreaves JR, Hayes RJ. Bias and inference from misspecified mixed-effect models in stepped wedge trial analysis. Stat Med. 2017;36(23):3670–82. 28556355 10.1002/sim.7348 PMC5600088

[53] Baio G, Copas A, Ambler G, Hargreaves J, Beard E, Omar RZ. Sample size calculation for a stepped wedge trial. Trials. 2015;16(1):354. 26282553 10.1186/s13063-015-0840-9 PMC4538764

[54] McCann RS, Messina JP, MacFarlane DW, Bayoh MN, Gimnig JE, Giorgi E, et al. Explaining variation in adult Anopheles indoor resting abundance: the relative effects of larval habitat proximity and insecticide-treated bed net use. Malar J. 2017;16(1):288. 28716087 10.1186/s12936-017-1938-1 PMC5514485

[55] Sangbakembi-Ngounou C, Costantini C, Longo-Pendy NM, Ngoagouni C, Akone-Ella O, Rahola N, et al. Diurnal biting of malaria mosquitoes in the Central African Republic indicates residual transmission may be “out of control.” Proc Natl Acad Sci U S A. 2022;119(21):e2104282119. 35576470 10.1073/pnas.2104282119 PMC9173762

[56] North AR, Burt A, Godfray HCJ. Modelling the suppression of a malaria vector using a CRISPR-Cas9 gene drive to reduce female fertility. BMC Biol. 2020. 10.1186/s12915-020-00834-z. 32782000 10.1186/s12915-020-00834-z PMC7422583

[57] Phuc HK, Andreasen MH, Burton RS, Vass C, Epton MJ, Pape G, et al. Late-acting dominant lethal genetic systems and mosquito control. BMC Biol. 2007;5(1):11. 17374148 10.1186/1741-7007-5-11 PMC1865532

[58] Multerer L, Glass TR, Vanobberghen F, Smith T. Analysis of contamination in cluster randomized trials of malaria interventions. Trials. 2021;22(1):613. 34507602 10.1186/s13063-021-05543-8 PMC8434732

[59] Utarini A, Indriani C, Ahmad RA, Tantowijoyo W, Arguni E, Ansari MR, et al. Efficacy of *Wolbachia*-infected mosquito deployments for the control of Dengue. N Engl J Med. 2021;384(23):2177–86. 34107180 10.1056/NEJMoa2030243 PMC8103655

[60] Vicente JL, Clarkson CS, Caputo B, Gomes B, Pombi M, Sousa CA, et al. Massive introgression drives species radiation at the range limit of *Anopheles gambiae*. Sci Rep. 2017;7(1):46451. 28417969 10.1038/srep46451 PMC5394460

[61] Hui T-YJ, Epopa PS, Millogo AA, Yao FA, Koulmaga D, Burt A, et al. Variance partition reveals contrasting random effect contributions on the density and species composition of malaria-transmitting mosquitoes. bioRxiv. 2025:2025.07.09.663957. 10.1186/s13071-026-07406-0 PMC13220501 42001154

[62] Santolamazza F, Mancini E, Simard F, Qi Y, Tu Z, della Torre A. Insertion polymorphisms of SINE200 retrotransposons within speciation islands of *Anopheles gambiae* molecular forms. Malar J. 2008;7(1):163. 18724871 10.1186/1475-2875-7-163 PMC2546427

[63] Rue H, Martino S, Chopin N. Approximate bayesian inference for latent Gaussian models by using integrated nested laplace approximations. J R Stat Soc Ser B Stat Methodol. 2009;71:319–92.

[64] Hancock PA. https://github.com/pahanc/Mosquito-suppression-trials/tree/main (10.5281/zenodo.17052718). 2025.

[65] Arnold BF, Hogan DR, Colford JM, Hubbard AE. Simulation methods to estimate design power: an overview for applied research. BMC Med Res Methodol. 2011;11(1):94. 21689447 10.1186/1471-2288-11-94 PMC3146952

[66] StataCorp. Stata Statistical Software: Release 18. College Station, TX: StataCorp LLC; 2023.

