# Supplementary material for: Requirements for designing cluster randomised control trials to detect suppression of malaria vector population densities
Source: BMC Biol. 2025 Oct 9;23:303. doi: 10.1186/s12915-025-02414-5 (PMC12512543; doi:10.1186/s12915-025-02414-5)
Supplement: Supplementary file 1 — Additional file 1: Fig. S1. The Delaunay triangulation mesh used in the SPDE model. Fig. S2. The number of compounds from which PSC collections were made per month across all months in Bana village, Bana market, Pala and Souroukoudingan. Fig. S3. The number of female mosquitoes genotyped for species identification in each village and month across all months in the period 2017-2019. Fig. S40. The distributions of the numbers of mosquitoes of each species collected per compound per month from each village. Fig. S5. The design of the step-wedge trial modelled in this study. Clusters shaded in blue receive the intervention and unshaded clusters do not receive the intervention. Fig. S6-S8. Posterior predicted mean counts of all An. gambiae complex species at each mesh node of the SPDE model across Bana village, Pala and Souroukoudingan. Fig. S9. Posterior predicted mean counts of all An. gambiae complex species for each month at ten randomly selected locations in Bana village, Pala, and Souroukoudingan. Fig. S10-S11. The effect sampling only during the rainy season (May-October) on the power to detect a suppression effect G=70% and 90%acting on all An. gambiae complex species (An. gambiae, An. coluzzii, and An. arabiensis). Fig. S12. The power to detect a suppression effect G acting on targeted vector species when no baseline data on mosquito counts is available. [file 12915_2025_2414_MOESM1_ESM.docx]

SUPPLEMENTARY MATERIAL

**“Requirements for designing cluster randomised control trials to detect suppression of malaria vector population densities”**

Penelope A. Hancock^*1^, Tin-Yu J. Hui^2^, Patric S. Epopa^3^, Azize Milogo^3,4^, Andrew R. McKemey^2^, Franck A. Yao^3^, Abdoulaye Diabaté^3^, Austin Burt^2^

Table of Contents

[1. Bayesian geostatistical model formulation 1](#_Toc207877050)

[2. Sample sizes and missing data in the PSC data set 3](#_Toc207877051)

[3. The design of the step wedge CRCT 7](#_Toc207877052)

[4. Predictive maps of mosquito counts 7](#_Toc207877053)

[5. Effects of rainy season-only sampling on power for other suppression effects 10](#_Toc207877054)

[6. Power to detect suppression without adjusting for baseline mosquito counts 12](#_Toc207877055)

## Bayesian geostatistical model formulation

The multivariate spatiotemporal Gaussian process, $f\left( \boldsymbol{x}_{h},m \right)$, is modelled assuming independent spatial and temporal autocorrelation structures. Spatial autocorrelation is modelled by a temporally uncorrelated Gaussian process, $\omega(\boldsymbol{x}_{h},m)$, with a variance $\sigma_{\omega}^{2}$ and a spatial correlation structure *C(h)* that is constant in time and defines the spatial dependence structure of the observations [37]. Thus,

$$Cov\left( \omega\left( \boldsymbol{x}_{h},m \right),\omega\left( \boldsymbol{x}_{h},m^{'} \right) \right)=\left\{ \begin{aligned} 0, m\neq m' \\ \sigma_{\omega}^{2}C\left( h \right) m=m' \end{aligned} \right.$$

Following the integrated nested Laplace approximation approach (INLA), $\omega(\boldsymbol{x}_{h},m)$ is approximated by a Gaussian Markov Random Field (GMRF) assuming that *C(h)* is a Matèrn covariance function [39]. In developing the INLA approach, Lindgren et al. [39] used the Matèrn covariance function to approximate a GMRF using stochastic partial differential equations. The Matèrn covariance function *C(h)* depends on the spatial separation distance between two points, $h=\left\| \boldsymbol{x}_{h}-\boldsymbol{x}_{h'} \right\|$, as well as a scale parameter $\kappa$ which controls the spatial autocorrelation range and a fixed smoothing parameter $\nu$ which is set to 1. The spatial process $\omega(\boldsymbol{x}_{h},m)$ evolves through time according to a first-order temporal autoregressive process (AR(1)) with a correlation $\phi$.

We used penalised complexity precision priors [40] for the standard deviations $\sigma_{\omega}$, $\sigma_{v}$ and $\sigma_{h}$. For $\sigma_{\omega}$ and $\sigma_{\nu}$ we set the upper limit to *U*=2 and the probability parameter to 0.1. For $\sigma_{h}$we set *U=*0.09 and the probability to 0.0001. For $\kappa$ we set a PC prior on the range parameter [38] setting the lower limit *U=*0.001 and the probability to 0.001.

*Defining the mesh for the SPDE model*

The mesh used in the R-INLA for the SPDE GMRF approximation of $f\left( \boldsymbol{x}_{h},m \right)$ was defined to have a fine resolution in the vicinity of the four villages (Bana, Bana Market, Pala and Souroukoudingan) and a coarse resolution throughout the remaining area encompassing the villages (Figure S1). The R-INLA commands for creating the mesh are provided in our Github repository (<https://github.com/pahanc/Mosquito-suppression-trials>).


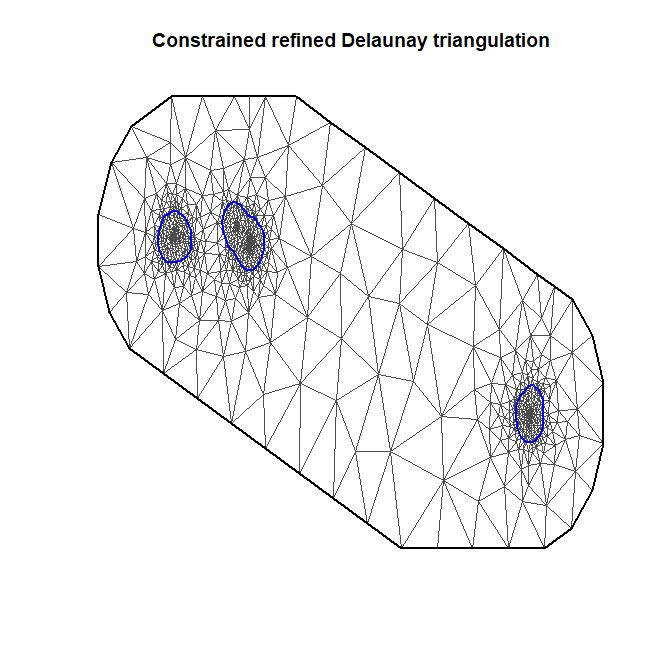


Figure S1. The Delaunay triangulation mesh used in the SPDE model.

## Sample sizes and missing data in the PSC data set

The space-time distribution of the PSC collections varied between months and years because ten compounds from each village (except Bana Market) were randomly selected for PSC sampling each month, and because data records are missing for some compounds (Figure S2). Our method for simulating counts $\tilde{y}_{hmv}^{s}$ across a single year used the sampling locations recorded in the PSC data set in each month for all months of a single year selected at random from the full set of years (2012-2014, 2017-2019). Each of the three years in our simulated trial data sets used a year selected at random without replacement from the full set of years. A different random selection of years was made for each cluster in a single simulated trial. There was no requirement for the selected years to be consecutive. We note that the choice of year affects the sampling locations and the number of missing samples but does not affect the value of the random effects $\mu_{v}$ and $\nu_{h}$.

**
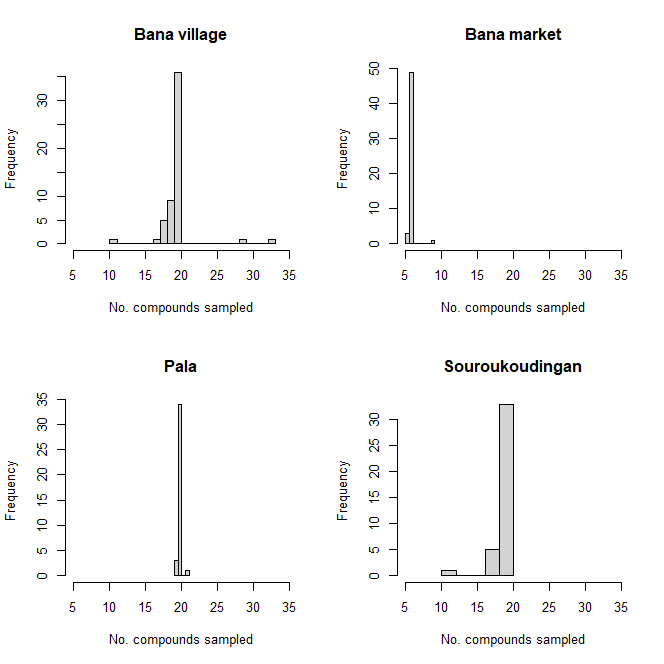
**

Figure S2. The number of compounds from which PSC collections were made per month across all months in Bana village, Bana market, Pala and Souroukoudingan.

*Subsets of mosquitoes genotyped for species identification*

The number of mosquitoes retained for species identification in each compound, month and village, $S_{hmv}$, and the species composition of these samples, varies across months and years in the PSC data (Figure S3). Our simulated counts of *An. coluzzii* and *An. gambiae*, $\tilde{c}_{hmv}^{s}$ and$\tilde{g}_{hmv}^{s}$ , across a single year used the sampling locations for which PSC subsets $S_{hmv}$ were retained in each month for all months of a single year selected at random from the full set of years (2017-2019). The recorded values of $S_{hmv}$ and species composition were used. Each of the three years in our simulated trial data sets used a single year selected at random without replacement from the set of years (2017-2019). A different random selection of years was made for each cluster in the simulated trial. There was no requirement for the selected years to be consecutive.

**
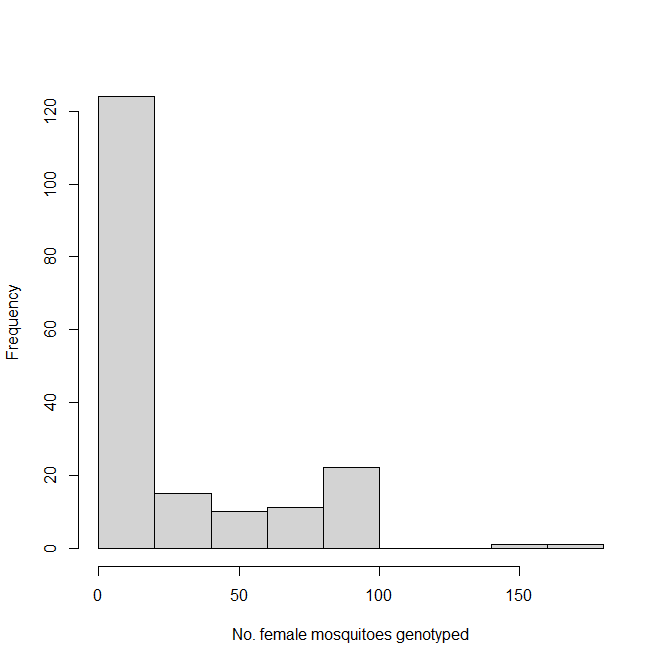
**

Figure S3. The number of female mosquitoes genotyped for species identification in each village and month across all months in the period 2017-2019.

**
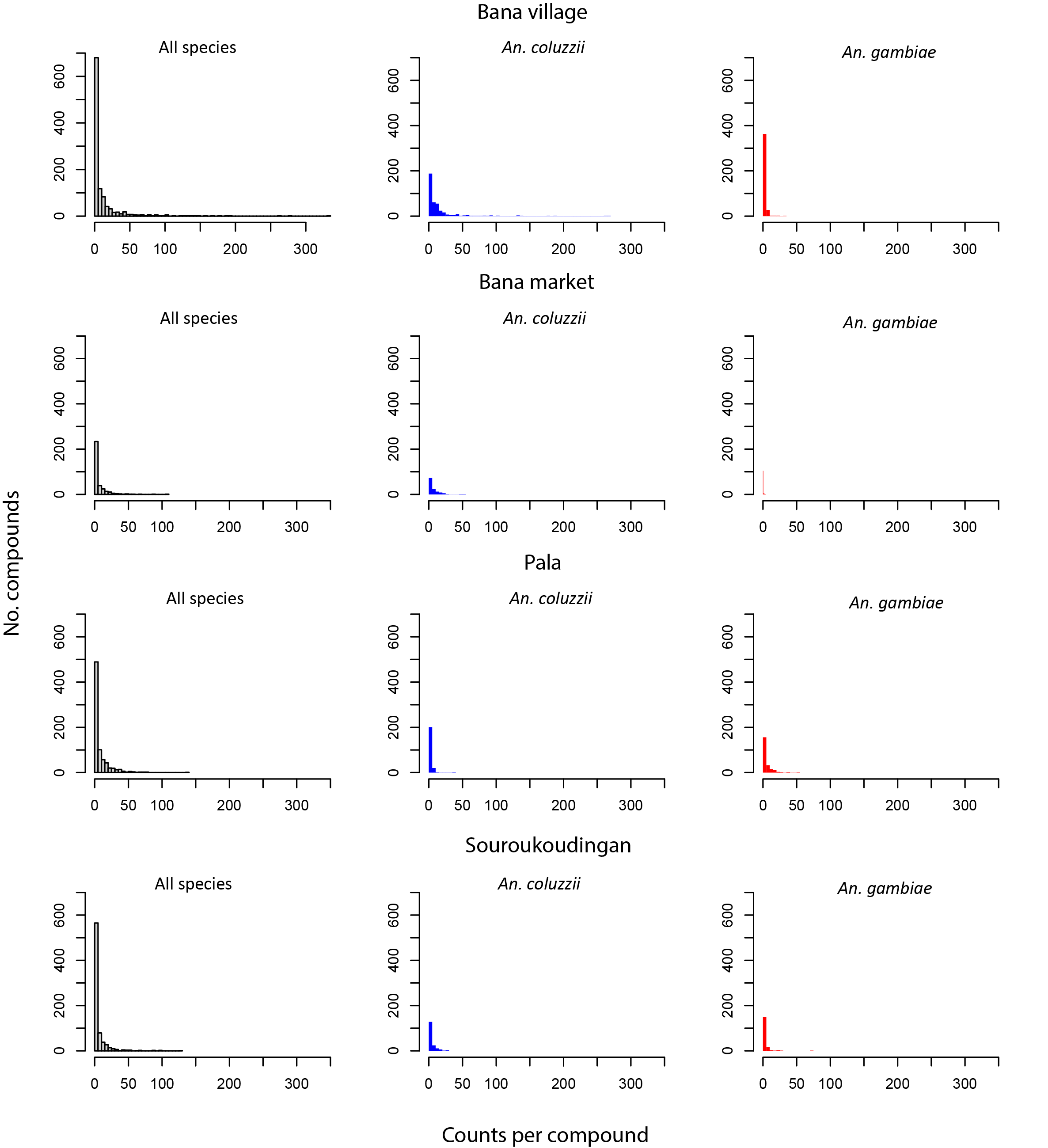
**

Figure S4. The distributions of the numbers of mosquitoes collected per compound per month from each village. Numbers of *An. gambiae* and *An. coluzzii* are estimates calculated by multiplying the number of mosquitoes of all species collected in a compound and month by the proportion of genotyped mosquitoes from that compound and month that were *An. gambiae* and *An. coluzzii*, respectively.

## The design of the step wedge CRCT

**
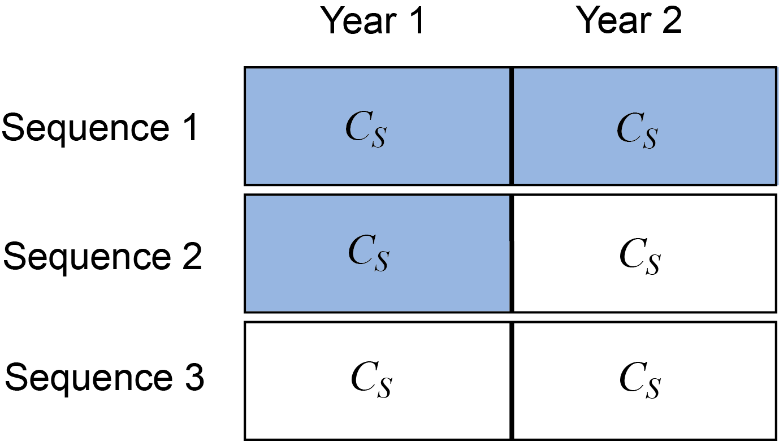
**

Figure S5. The design of the step-wedge trial modelled in this study. Clusters shaded in blue receive the intervention and unshaded clusters do not receive the intervention.

## Predictive maps of mosquito counts


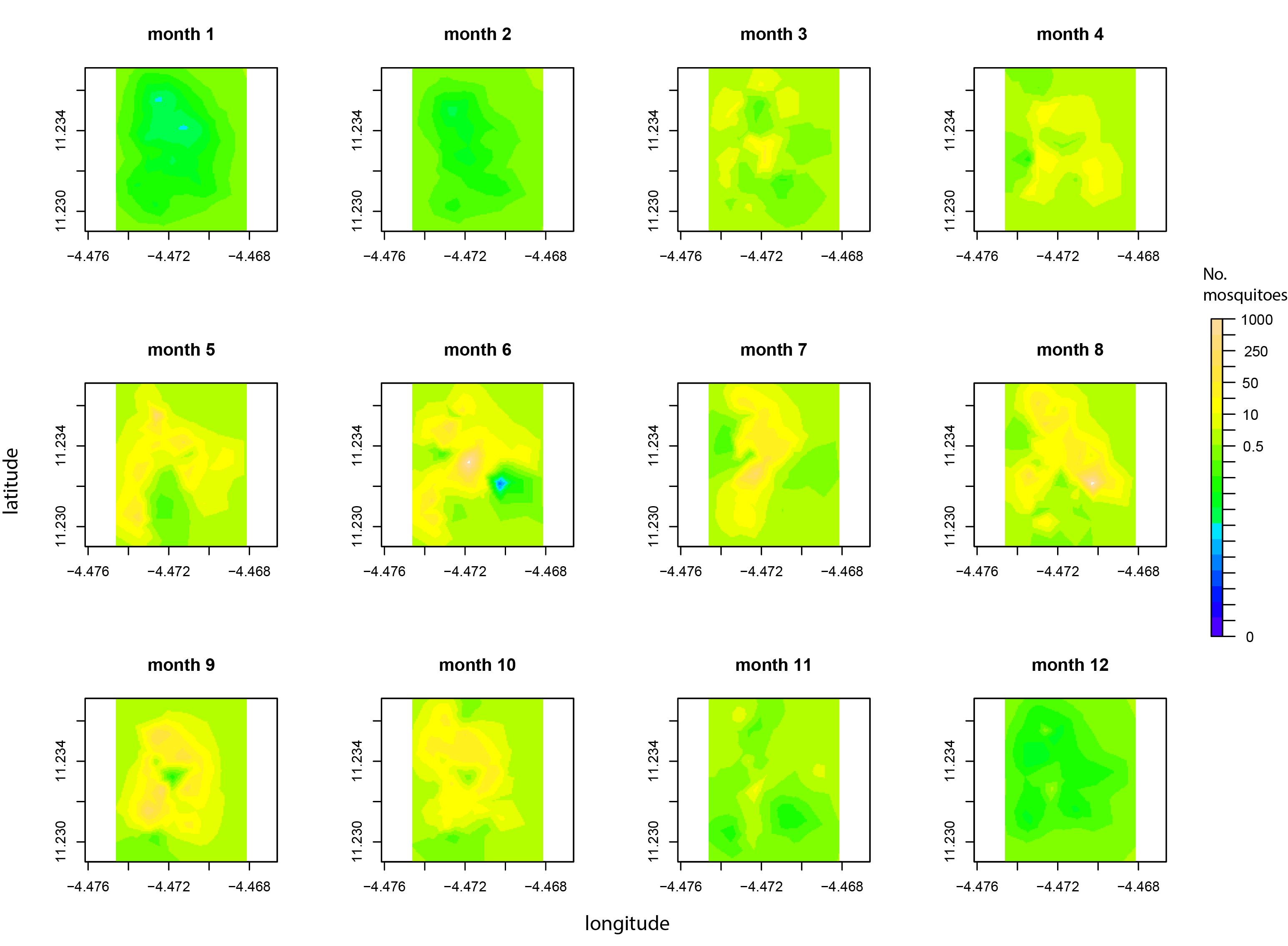


Figure S6. Posterior predicted mean counts of all *An. gambiae* complex species at each mesh node of the SPDE model across Bana village.


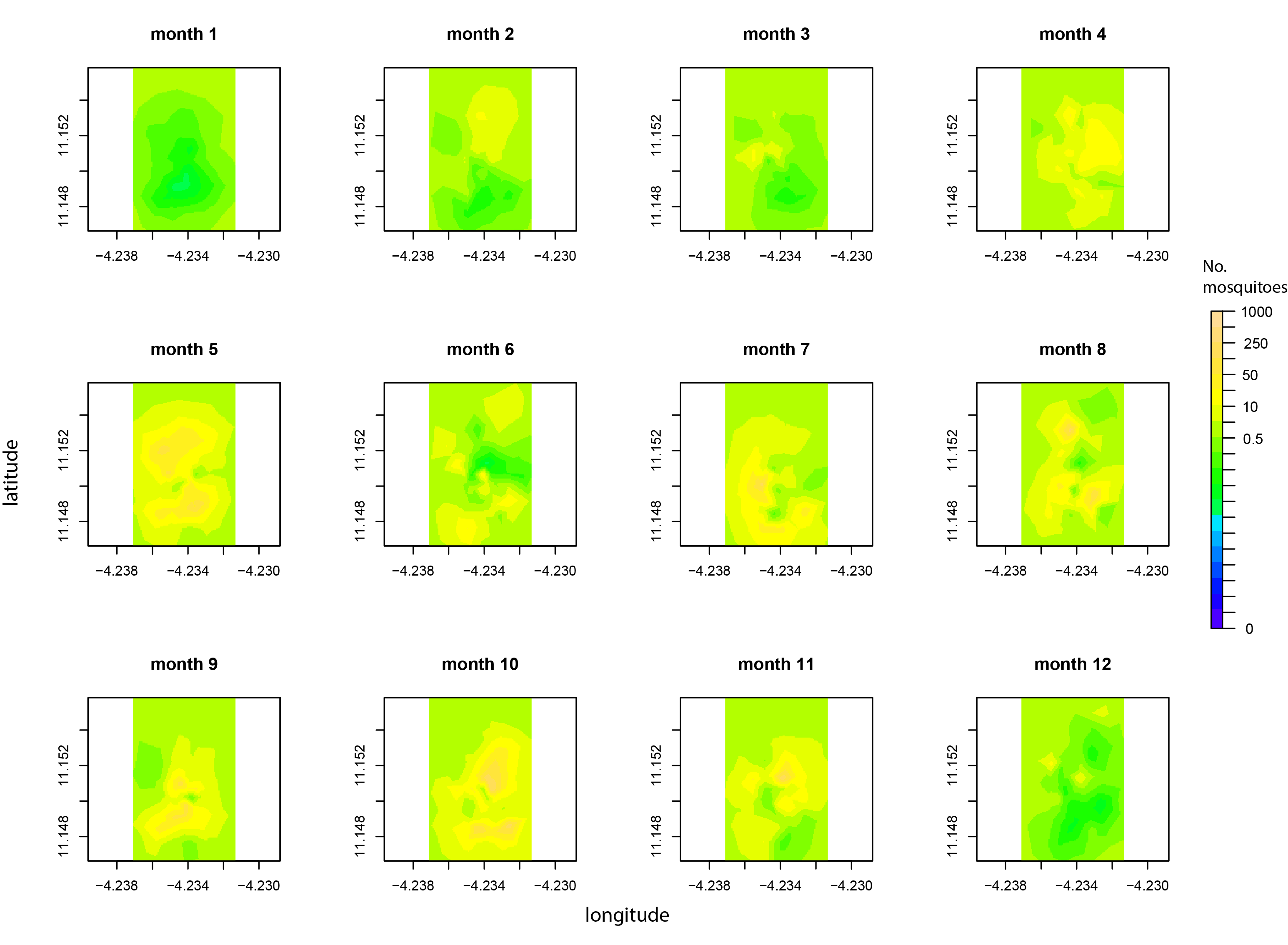


Figure S7. Posterior predicted mean counts of all *An. gambiae* complex species at each mesh node of the SPDE model across Pala.


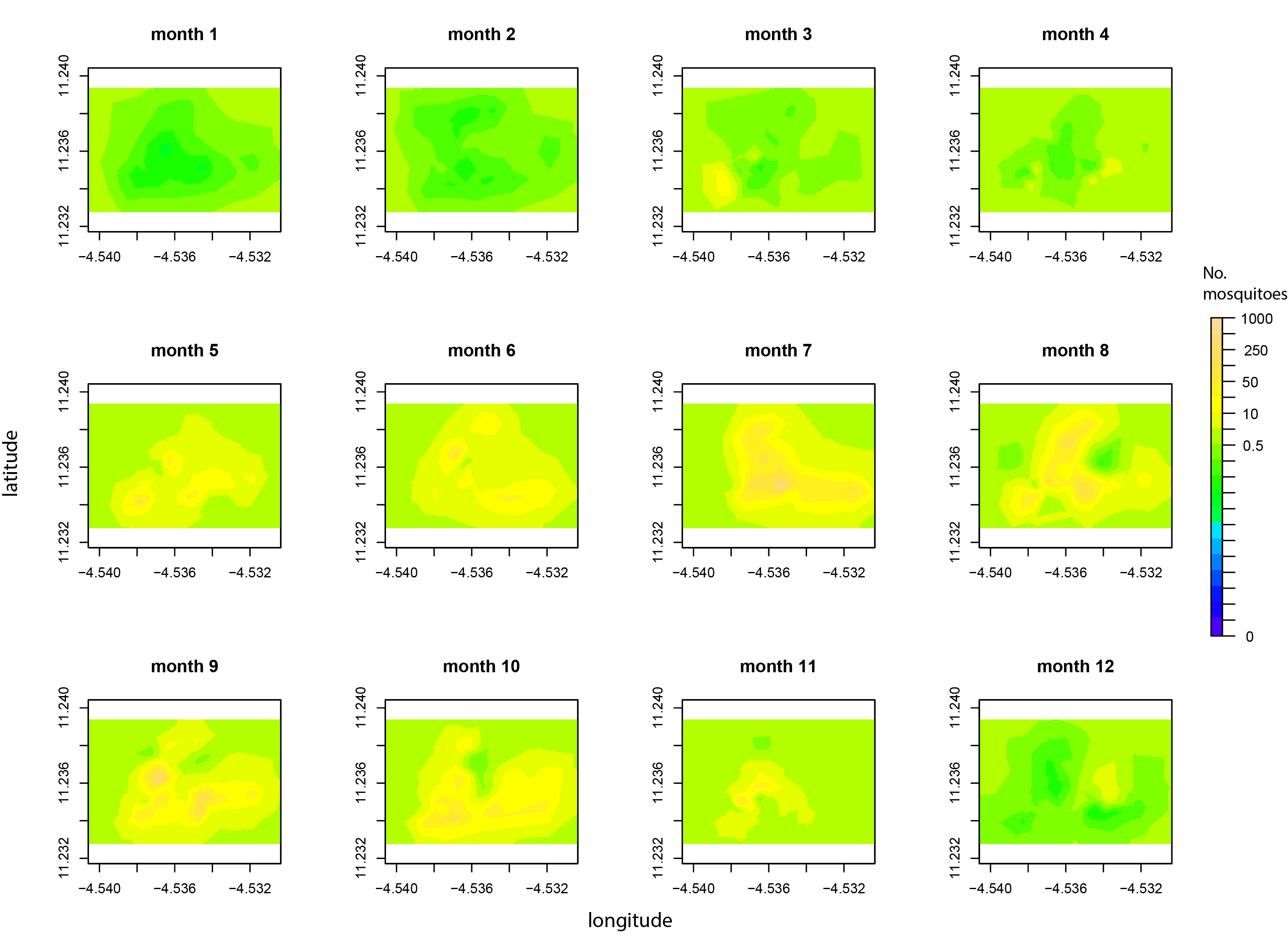


Figure S8. Posterior predicted mean counts of all *An. gambiae* complex species at each mesh node of the SPDE model across Souroukoudingan.


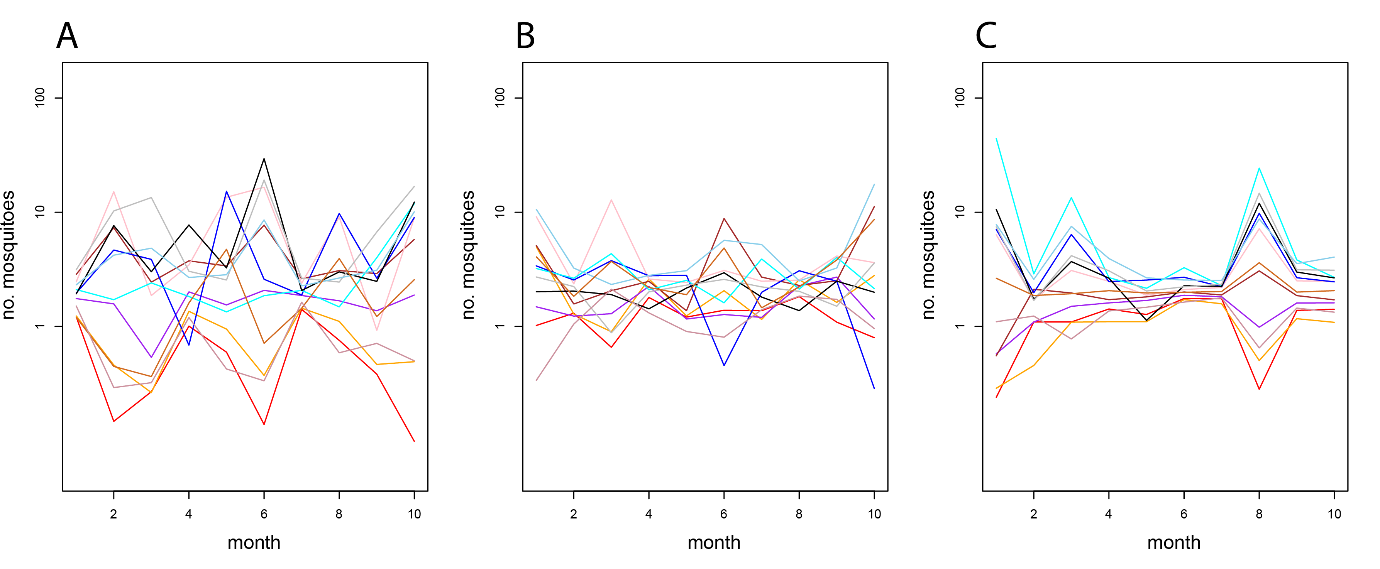


Figure S9. Posterior predicted mean counts of all *An. gambiae* complex species for each month at ten randomly selected locations in A. Bana village, B. Pala, and C. Souroukoudingan. Lines of each colour show predictions for a single location.

## Effects of rainy season-only sampling on power for other suppression effects

**
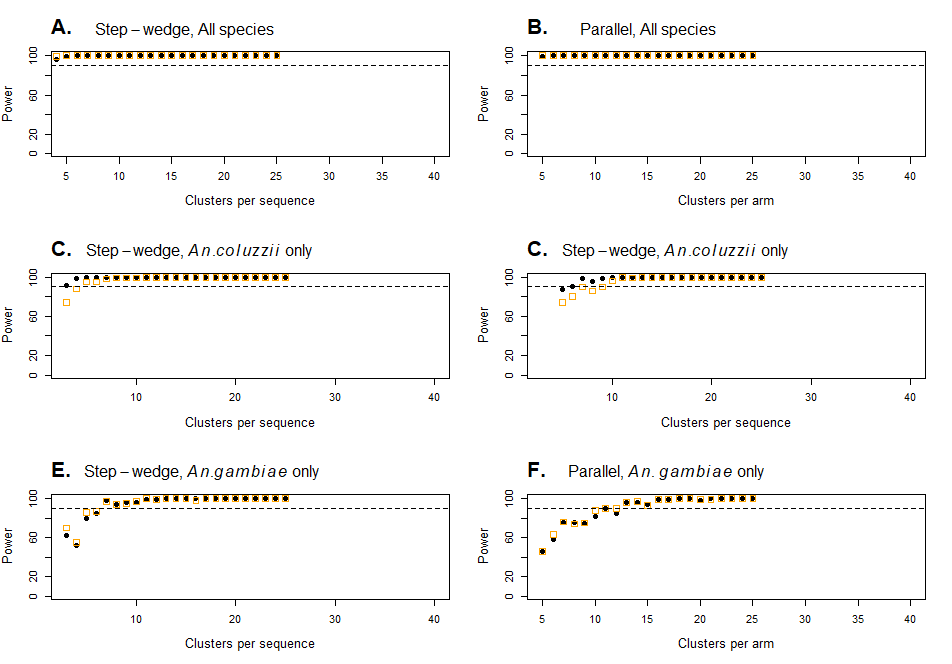
**

Figure S10. The effect sampling only during the rainy season (May-October) on the power to detect a suppression effect *G* acting on all *An. gambiae* complex species (*An. gambiae*, *An. coluzzii*, and *An. arabiensis*). Power values when collections were made in all months of the year (black circles), and only the rainy season months (yellow squares) are shown. Results for suppression effects of *G*=70% are shown. Dotted lines show the 90% power threshold. Rows show results when suppression affects all vector species (A,B), *An. coluzzii* only (C,D), and *An. gambiae* only (E,F). Both step wedge (column A,C,E) and parallel (column B,D,F) designs were modelled.

**
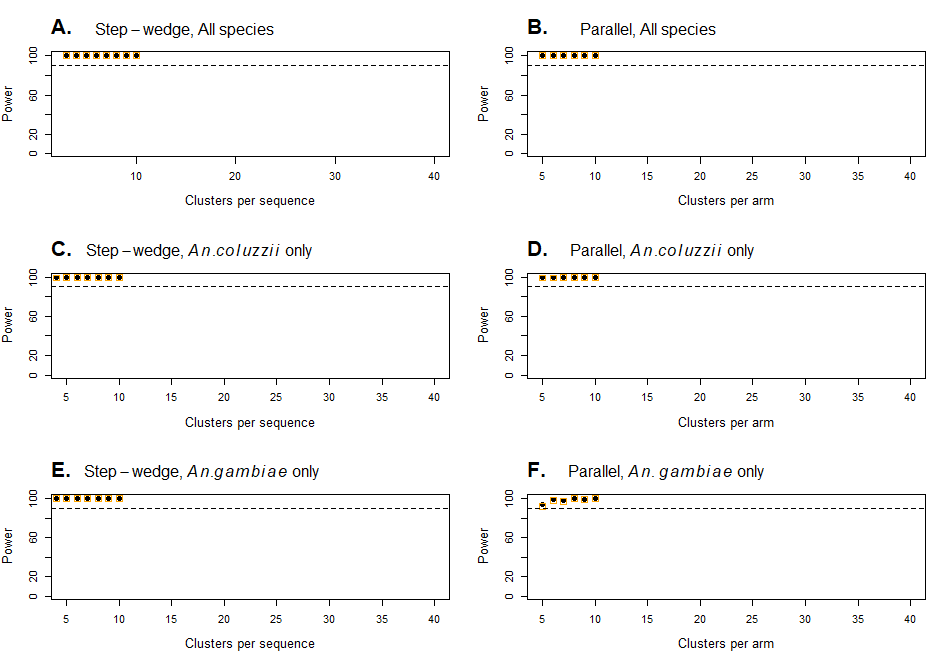
**

Figure S11. The effect sampling only during the rainy season (May-October) on the power to detect a suppression effect *G* acting on all *An. gambiae* complex species (*An. gambiae*, *An. coluzzii*, and *An. arabiensis*). Power values when collections were made in all months of the year (black circles), and only the rainy season months (yellow squares) are shown. Results for suppression effects of *G*=90% are shown. Dotted lines show the 90% power threshold. Rows show results when suppression affects all vector species (A,B), *An. coluzzii* only (C,D), and *An. gambiae* only (E,F). Both step wedge (column A,C,E) and parallel (column B,D,F) designs were modelled.

## Power to detect suppression without adjusting for baseline mosquito counts


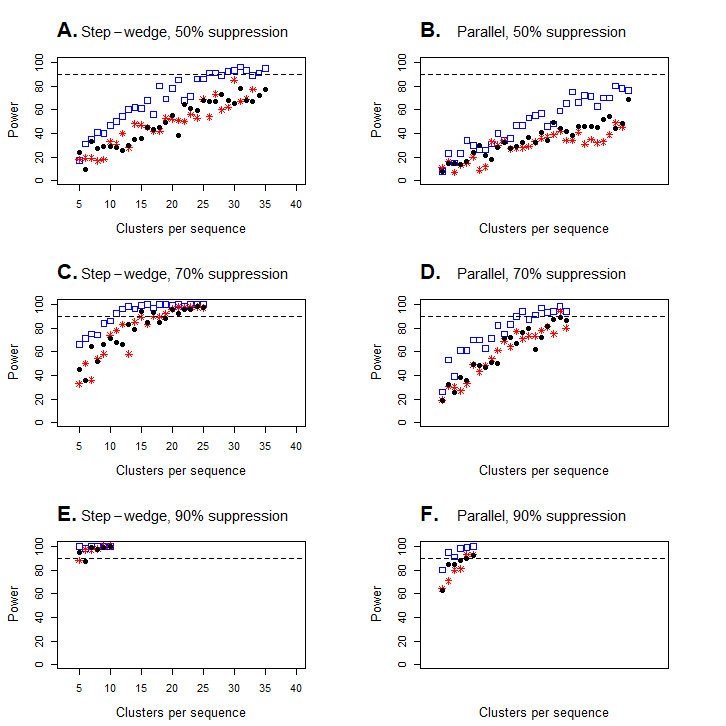


Figure S12. The power to detect a suppression effect *G* acting on targeted vector species when no baseline data on mosquito counts is available. Blue square markers show the power when all *An. gambiae* complex species (*An. gambiae*, *An. coluzzii*, and *An. arabiensis*) experience the suppression effect. Red asterisks and black circles show power values when only *An. coluzzii* (red markers) or only *An. gambiae* (black markers) is suppressed. Dotted lines show the 90% power threshold. Suppression effects of 50% (row A-B), 70% (row C-D) and 90% (row E-F) are shown. Both step wedge designs (column A,C,E) and parallel designs (column B,D,F) were modelled.
